# Supporting Meaningful Choices: A Decision Aid for Individuals Facing Existential Distress and Considering Psilocybin-Assisted Therapy

**DOI:** 10.3390/healthcare13182290

**Published:** 2025-09-12

**Authors:** Ariane Bélanger, Sue-Ling Chang, Jean-François Stephan, Florence Moureaux, Diane Tapp, Robert Foxman, Pierre Gagnon, Johanne Hébert, Houman Farzin, Michel Dorval

**Affiliations:** 1 Faculty of Pharmacy, Université Laval, Québec, QC G1V 0A6, Canada; ariane.belanger.7@ulaval.ca; 2 CHU de Québec-Université Laval Research Center, Oncology Program, Québec, QC G1S 4L8, Canada; sue-ling.chang@crchudequebec.ulaval.ca (S.-L.C.); diane.tapp@fsi.ulaval.ca (D.T.); pierre.gagnon@crchudequebec.ulaval.ca (P.G.); 3 Réseau Québécois de Recherche en Soins Palliatifs et de Fin de Vie (RQSPAL), Québec, QC G1J 1Z4, Canada; johanne_hebert@uqar.ca; 4 Private Practice, Montréal, QC H2C 1E3, Canada; jf.stephan@outlook.com; 5 Patient-Partner, Québec, QC, Canada; floreaux@icloud.com (F.M.); rhfoxman@gmail.com (R.F.); 6 Faculty of Nursing, Université Laval, Québec, QC G1V 0A6, Canada; 7 Équipe de Recherche Michel-Sarrazin en Oncologie Psychosociale et Soins Palliatifs, Québec, QC G1V 0A6, Canada; 8 Faculty of Medicine, Université Laval, Québec, QC G1V 0A6, Canada; 9 CISSS de Chaudière-Appalaches Research Center, Lévis, QC G6V 3Z1, Canada; 10 Department of Health Sciences, Université du Québec à Rimouski, Lévis, QC G6V 0A6, Canada; 11 Lady Davis Institute, Jewish General Hospital, Montréal, QC H3T 1E2, Canada; houman.farzin@mcgill.ca; 12 Faculty of Medicine and Health Sciences, McGill University, Montréal, QC H3G 2M1, Canada

**Keywords:** decision aids, psilocybin-assisted therapy, existential distress, palliative care, end of life

## Abstract

**Background/Objectives**: Given the limitations of traditional approaches to treating existential distress in seriously ill patients, psilocybin-assisted therapy (PAT) has emerged as a promising treatment option. However, weighing up the potential risks and benefits of this approach can be challenging for both healthcare professionals and patients. Decision aids can play a key role in supporting shared decision making by clarifying options, improving knowledge, and enhancing decision quality. To date, there is no decision aid specific to PAT. This descriptive study aimed to develop a decision aid for individuals considering this therapy. **Methods**: A paper-based/electronic decision aid was developed with a multidisciplinary steering committee following the International Patient Decision Aids Standards Collaboration (IPDAS). Development included conducting a literature review and prototype design, evaluating acceptability and usability by potential users (i.e., patients and healthcare professionals), and producing a final version. Questionnaires, direct feedback, and semi-structured interviews with potential users allowed for evaluation and refinement of design and content. **Results**: The final version of the decision aid is presented as a booklet, covering areas such as PAT education, comparison of treatment options, and personal reflection. Feedback from patients (*n* = 5) and healthcare professionals (*n* = 5) guided improvements, helping clarify content, ensuring balanced information, optimizing its length for usability, and providing decision-making support. **Conclusions**: The decision aid developed in this study demonstrated satisfactory acceptability and usability, meeting IPDAS criteria. By providing balanced and accessible information, it may facilitate shared decision-making for individuals considering PAT, representing a significant step forward in this emerging area of palliative care.

## 1. Introduction

Being diagnosed with a potentially fatal and incurable illness can trigger profound existential distress, impacting the physical, emotional, social, and spiritual well-being of individuals [1]. Existential distress may manifest as a loss of meaning and dignity, suicidal ideation, death-related anxiety, and heightened pain perception, often accompanied by symptoms of anxiety, depression, and demoralization [2,3]. It is estimated that approximately one-third of patients with advanced cancer experience such distress, which compromises their quality of life and their ability to experience a meaningful end-of-life period [4,5].

Although various psychosocial interventions have been developed, their effectiveness and feasibility in treating existential distress remain limited. Conventional pharmacological treatments, such as antidepressants and anxiolytics, have shown marginal effects [6,7]. These limitations have led to growing interest in innovative approaches to address existential distress, including psilocybin-assisted therapy (PAT). Clinical trials have demonstrated that psilocybin, combined with psychotherapy, can significantly reduce symptoms of anxiety and depression in patients with life-threatening cancer, with effects lasting at least six months in up to 80% of selected patients [8,9]. Systematic reviews have similarly supported the findings of PAT as an effective intervention for existential distress [10,11,12]. These promising results offer an alternative to the shortcomings of conventional treatments.

Internationally, various jurisdictions have begun integrating PAT into their healthcare systems [13,14,15,16]. Given the growing medical interest and the progressive lifting of legislative barriers, more individuals are expected to seek PAT in the coming years. As a relatively new approach, PAT raises important questions, and many people may lack the knowledge needed to determine if it is a viable option. Given this, this approach requires a thorough evaluation of risks and benefits, as well as consideration of patients’ personal values [17]. To our knowledge, no patient decision aid currently exists to help individuals considering PAT weigh its potential benefits and risks within a structured shared decision-making process. While we identified various information resources (e.g., brochures, guides, podcasts, documentaries), these do not meet the IPDAS definition of a patient decision aid. Decision aids aim to make the decision explicit, provide information about the options and their potential health outcomes, and clarify the individual’s personal values regarding the therapeutic option [18]. Their use is particularly recommended in situations where no single therapeutic option stands out as superior, which perfectly aligns with the context in which PAT may be considered [18,19]. Decision aids have been applied in mental health care, where they may improve knowledge, reduce decisional conflict, and support value-congruent choices; some studies also suggest potential benefits for psychological outcomes [20,21]. These findings suggest the relevance of developing a structured decision aid for patients considering psilocybin-assisted therapy. This study aimed to develop a decision aid tool for individuals considering PAT to alleviate existential distress. The specific objectives were to (1) design a decision aid tool and (2) assess its acceptability and usability among potential users.

## 2. Materials and Methods

**Figure 1:**
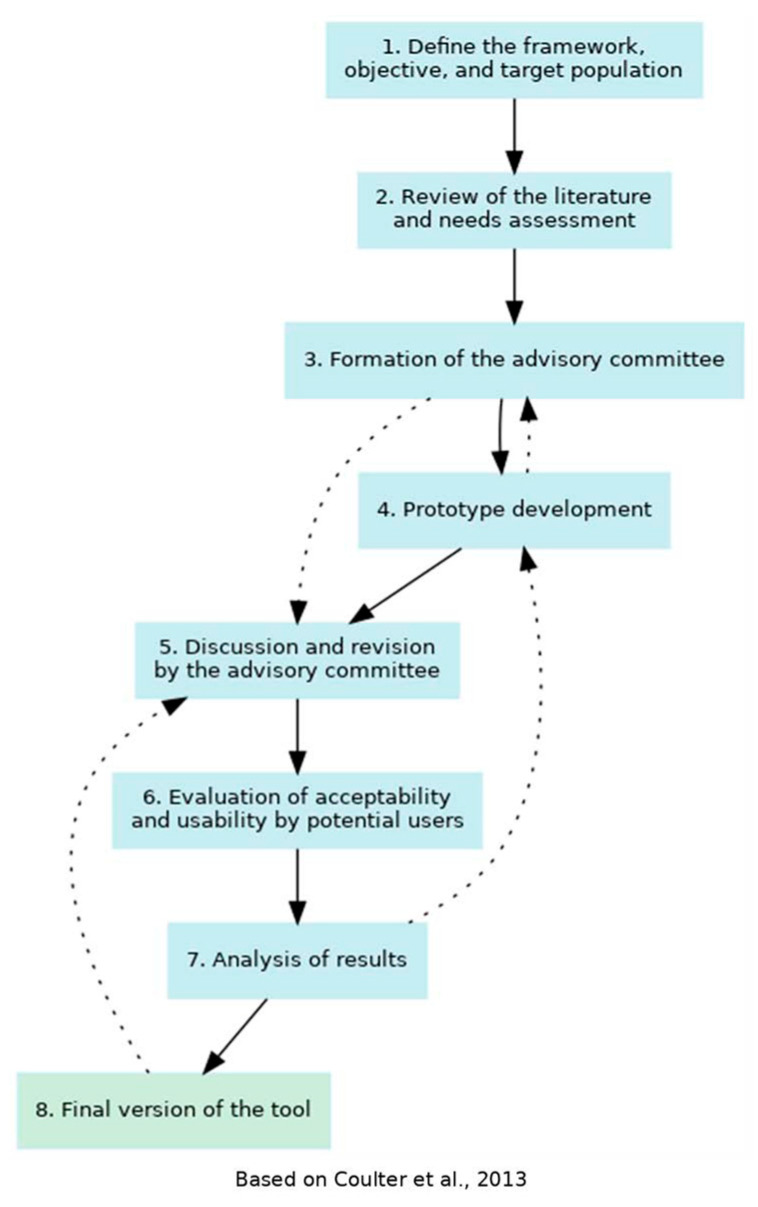
Decision aid development process, [25].

The decision aid was developed in accordance with the criteria of the *International Patient Decision Aids Standards Collaboration* (IPDAS) [22] and the *Ottawa Decision Support Framework* [23]. The methodology was divided into two main phases: the design of the prototype and the evaluation of its acceptability and usability. The adapted 8-step IPDAS model guided the development of the decision aid (see Figure 1). In addition, the reporting of this study was guided by the SUNDAE (Standards for Universal Reporting of Patient Decision Aid Evaluation) checklist, which aims to enhance the quality and transparency of research on patient decision aids [24] (see Supplementary File S1).

### 2.1. Prototype Development

A critical analysis of the benefits and risks of PAT was conducted based on a literature review. Drawing on the available scientific evidence, a preliminary version of the prototype was developed through an iterative process by a multidisciplinary steering committee comprising researchers, physicians, nurses, and patient partners. Each iteration was carefully reviewed and edited until consensus was reached. Revising the content and selecting appropriate graphics were key steps in the development process. The prototype was refined to meet acceptability and usability criteria based on clarity, balanced information, and the use of the best available evidence [18,25,26,27,28].

Recognizing the impact of health literacy on decision-making [29], the decision aid was designed with a focus on language accessibility. The Flesch-Kincaid readability test (FK) was used to measure reading ease, targeting a score suitable for the broadest possible audience [30]. We combined this test with evaluations from potential users to ensure optimal clarity and comprehension [31]. The occasional use of ChatGPT (GPT-4o) helped simplify certain sections to achieve a reading level equivalent to or below the 8th grade, corresponding to a Flesch-Kincaid score of 8 [18,25,26,27,28]. To ensure clarity, medical accuracy, and neutrality, a final literacy validation was conducted by the multidisciplinary steering committee, which included researchers, physicians, nurses, and patient partners.

### 2.2. Evaluation of Prototype Acceptability and Usability

This qualitative aspect of the study is reported according to the Consolidated Criteria for Reporting Qualitative Research (COREQ) guidelines (see Supplementary File S2).

#### Sampling and Participant Recruitment

A purposive sampling strategy was employed to recruit potential decision aid users (e.g., individuals living with serious and incurable illnesses—referred to here as “patients” for the sake of simplicity—and healthcare professionals) who were not involved in the steering committee. This approach was chosen to include a diverse and representative range of perspectives from both groups. Patients were recruited via institutional mailing lists and social media, while healthcare professionals were identified through the authors’ professional networks. All participants were required to have sufficient proficiency in French to complete the questionnaires and interviews. Those who agreed to participate received the consent form, the prototype, and detailed instructions for scheduling an interview via email, ensuring informed and structured participation in the study.

### 2.3. Data Collection

#### 2.3.1. Reading Grid and Sociodemographic Questionnaire

A reading grid, adapted from the work of O’Connor and Cranney [32] was developed to allow standardized prototype evaluation by the steering committee and research participants (see Supplementary File S3). This grid evaluated the prototype based on acceptability and usability criteria, including content quality, presentation clarity, comparison with other treatment options, and support for decision making [32] using a 4-point scale from “not at all satisfactory” to “very satisfactory”, with a comment section for suggestions and impressions. A self-administered questionnaire was also used to collect sociodemographic and occupational data.

#### 2.3.2. Semi-Structured Interviews

Individual interviews were conducted with participants to gather their thoughts and impressions on the prototype. Inspired by cognitive interviewing methods [33,34,35,36], participants were invited to comment on elements such as titles and subtitles, section order, content, and the clarity of the information presented. The interview guide followed IPDAS quality criteria and was adapted from existing tools used to evaluate decision aids [18,28,37,38]. It was reviewed by the steering committee and further refined iteratively as themes emerged during data collection (see Supplementary File S4).

Interviews were conducted in person or remotely via Teams or Zoom between March and September 2024. All interviews were conducted by a female graduate student and palliative care nurse who was experienced with conducting qualitative interviews (AB) with no other person present. Interviews lasted approximately one hour, were audio recorded, and fully transcribed. Field notes were taken during each session to capture contextual observations, the researcher’s initial impressions, and participants’ reactions. These notes enriched the analysis by providing additional data not captured in audio recordings. A reflective journal was maintained throughout data collection to document the researcher’s thoughts on the interview process, potential biases, and the evolution of the research.

### 2.4. Data Analysis

Using data from the reading grid, transcriptions, and participants’ comments, the first author (A.B.) conducted a thematic synthesis of the feedback based on the topics discussed during the interviews. Transcripts were coded by a single coder (A.B.) with ongoing discussion regarding emerging themes (M.D.). The results of this synthesis were then reviewed with steering committee members to reach a consensus on the changes to be made to the prototype to develop the final version. Qualitative data were analyzed using NVivo 15 software, allowing for systematic coding of the interview transcripts and structuring emerging themes.

### 2.5. Ethical Considerations

This study was approved by the Research Ethics Committee of CHU de Québec–Université Laval (2024-7116). Written informed consent was obtained from all participants. Data were collected anonymously and used solely for research purposes. Results were aggregated, ensuring no participant could be identified. All participants received monetary compensation for their time (50 CAD).

## 3. Results

### 3.1. Prototype Development

A booklet was developed titled “Envisager la thérapie assistée par psilocybine pour soulager la détresse existentielle/Psilocybin-assisted therapy for existential distress: Making the right choice for you” with the steering committee. The decision aid featured four key sections to support informed decision-making regarding PAT. One section provided educational information on existential distress and PAT, outlining potential benefits, risks, contraindications, and possible drug interactions. Another offered an objective comparison of therapeutic options, such as medications, psychotherapy, spiritual interventions, and PAT, helping patients understand how each approach fits within the broader spectrum of available treatments for existential distress. The prototype also encouraged personal reflection on values and care priorities to support decisions that align with individual goals [19]. The document included visual elements, a values clarification scale, and a frequently asked questions (FAQ) section to enhance understanding and user engagement. These four sections were selected based on IPDAS standards, prior research on shared decision making, and feedback from patients and healthcare professionals, highlighting the need for balanced information, comparison of options, values clarification, and accessible formats.

### 3.2. Evaluation of Acceptability and Usability

#### Participant Characteristics

Data from 5 patients and 5 healthcare providers were collected. All recruited participants completed the interviews; no dropouts occurred. Patients were mostly women (*n* = 4) and aged 55 and over (*n* = 3). Among these potential users, three were living with cancer and two had a degenerative condition. Their level of education was high, with all having completed undergraduate or graduate university studies (see Table 1). The healthcare professionals, mostly male, included three family physicians and two psychiatrists.

### 3.3. Evaluation Using the Reading Assessment Grid

**Figure 2:**
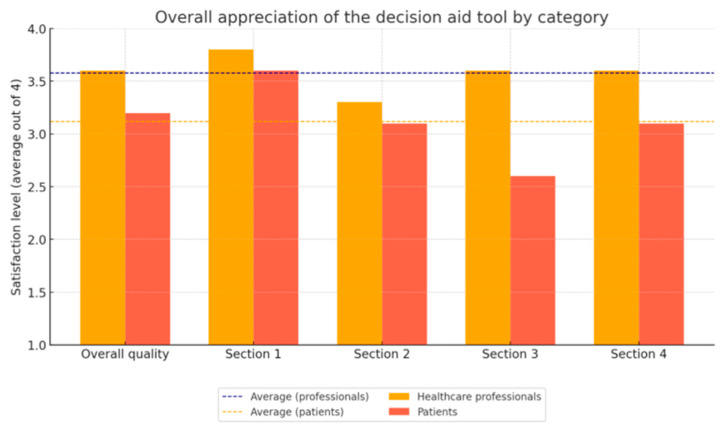
Level of participant satisfaction.

The evaluation focused on the booklet’s overall quality and four key sections of the decision aid, each rated on a satisfaction scale ranging from 1 (not at all satisfactory) to 4 (very satisfactory). The overall quality of the decision aid was considered satisfactory, with an average score of 3.2 from patients and 3.6 from healthcare professionals, indicating a generally positive perception (see Figure 2).

Section 1 of the decision aid aimed to expand knowledge about PAT within the context of existential distress and received an average rating of 3.4 from both groups. Section 2 compared PAT with other available treatment options and was rated 3.2 by all. Section 3, which encouraged reflection on personal values and care priorities, received a higher mean score from professionals (3.6) than from patients (2.6). This discrepancy may reflect differences in familiarity and comfort with structured values clarification tools. Patients may have perceived the section as less intuitive or more demanding, while professionals, accustomed to such frameworks, rated it more positively. These results suggested that clearer guidance and concrete examples were needed to optimize patient engagement with this section (see Figure 2).

Overall, these results point to high satisfaction, especially among healthcare professionals, with some areas for improvement. Section 3 was noted explicitly as needing revision due to differences in perception between the two groups. Based on participant feedback, this section was revised to better address user needs and improve the clarity and usefulness of the values clarification process.

### 3.4. Thematic Analysis of the Semi-Structured Interviews

Thematic analysis of the semi-structured interviews revealed several key themes that informed revisions to the decision aid tool (see Table 2, Table 3 and Table 4). These included the balance of options presented, the content, the clarity of the information, the length and structure of the document, and the most appreciated features.

- Theme 1: Balance of options

Regarding the balance of options, participants noted a perceived bias in favor of PAT, which they felt could influence patients’ decision making. As illustrated by healthcare professionals’ quotes (Table 3), some stressed the importance of including information on other therapeutic approaches, such as existential psychotherapy, ketamine-assisted therapy, and spiritual interventions. Several patients also noted the disproportionate focus on PAT (Table 2), although many acknowledged the limited availability of effective alternatives. The content was thus revised to better balance the presentation of available treatment options (Table 4).

- Theme 2: Content

The content of the decision aid itself also elicited varied reactions. Healthcare professionals highlighted the importance of clearly presenting PAT as an experimental option, mainly intended for refractory cases. Some also mentioned the risk of temporary symptom exacerbation following psilocybin administration (Table 3). Patients appreciated the description of existential distress but expressed concerns about specific terminology, particularly the phrase “large dose,” which was perceived as anxiety-provoking (Table 2). To address these concerns, a preamble clarifying the experimental nature of PAT was added, sensitive terms were reformulated, and the risks and benefits associated with the therapy were better contextualized (Table 4).

- Theme 3: Information clarity

The clarity of the information and health literacy were also central concerns. Although the decision aid tool was generally well received, several participants brought up difficulties in understanding certain concepts or visual elements. Healthcare professionals recommended simplifying certain complex medical terms, while patients pointed out confusion regarding the values clarification scale and comparative tables (Table 2 and Table 3). In response, the language was simplified, visuals were revised, and the comparative table was redesigned to improve readability and relevance (Table 4).

- Theme 4: Length and structure

Opinions were mixed regarding the length of the decision aid and the order of the sections. While some participants considered the content comprehensive and relevant, others reported cognitive fatigue when reading the material and suggested that the document could better balance information richness and accessibility. Participants also proposed reordering some sections to facilitate navigation (Table 2 and Table 3). As a result, the sequence of sections was revised, with the FAQ section moved before the values clarification scale, and some less relevant elements were removed (Table 4).

- Theme 5: Most appreciated features

Several aspects of the decision aid tool were unanimously appreciated. Both patients and healthcare professionals highlighted the relevance of the holistic approach, which addresses psychological, existential, and spiritual dimensions. They also appreciated the sections allowing personal questions to be noted and the detailed information on contraindications and drug interactions (Table 2 and Table 3). Overall, the decision aid was perceived as a useful and valuable resource to support patients in a thoughtful and informed decision-making process, provided it is adapted to the users’ practical needs and clinical contexts.

### 3.5. Final Version of the Decision Aid

Following the qualitative evaluation, a final version of the decision aid was produced and validated by the steering committee. This version included key modifications intended to improve readability, balance the presentation of treatment options, elucidate the values clarification scale, and enhance the overall accessibility and utility of the information. The structure of the booklet, organized into four main sections, was optimized to facilitate navigation and support personal reflection. Although we re-presented the final version to participants, only 2 patients and 1 healthcare professional completed the evaluation grid. Due to the patients’ fragile health, we did not recontact them further. Given the small sample size, we do not report post-evaluation statistics. However, qualitative feedback indicates that the final version was improved and generally perceived as clear, balanced, and useful. One patient mentioned: “I appreciate its ease of use and understanding, and the fact that it can be available at home to return to when needed. The booklet is, in my opinion, a good introduction and a helpful support. I don’t really have (any suggestions for improvement) at this stage.” A doctor said: “Very well explained; clear vocabulary that does not require prior knowledge, risks and benefits clearly presented, use of decision-making support, tables and figures that strongly support the content”.

Following the initial development of the decision aid in French, a North American English version was produced and revised by a patient-partner and a healthcare professional, both native English speakers involved in PAT, to ensure clarity, accuracy, and relevance for English-speaking users. For additional rigor, we also assessed readability using the Flesch-Kincaid scale to maintain consistency with the French version, which was required to achieve a score between 7 and 8; the English version reached 7.6, exactly within the recommended target for decision aids. Both versions are available in Supplementary Files S5 and S6.

The final decision aid meets the quality criteria established by IPDAS and was deemed relevant, balanced, and useful by participants in supporting decision making for individuals facing existential distress [19,22,26,27,39].

## 4. Discussion

The decision aid developed in this study is the first, to our knowledge, designed to support consideration of PAT for existential distress in the context of a life-threatening illness. The process was guided by a robust theoretical framework based on IPDAS and the Ottawa Decision Support Framework, both of which provided a strong methodological foundation aligned with current best practices. The development process was overseen by a multidisciplinary steering committee composed of researchers, clinicians, and patients with lived experience receiving PAT. The decision aid was informed by a literature review and refined with iterative rounds of feedback from the steering committee, and participant input. The decision aid developed demonstrated promising results regarding acceptability and usability among healthcare professionals and patients.

The overall high level of satisfaction reflects the decision aid’s relevance in supporting decision making for patients considering PAT. It meets several quality criteria defined by IPDAS, including the balanced presentation of options, the clear identification of the decision to be made, the description of risks and benefits for each option, and the inclusion of a values clarification process. These elements strengthen the decision aid’s validity within a shared decision-making framework in palliative care [19,22,26,27,40].

Nevertheless, the values clarification section emerged as an area requiring further refinement to optimize the decision aid’s clinical relevance. Divergences in satisfaction levels between patients and healthcare professionals regarding this section suggest a greater need for personalization in the decision-making process, highlighting the critical role of individual values in guiding treatment choices [27]. This issue was addressed in the final version of the decision aid tool, which includes a revised and more flexible values clarification component designed to better reflect individual priorities. In palliative care, aligning treatment decisions with patients’ personal goals and life priorities remains a cornerstone of high-quality care [28,41,42].

### 4.1. Comparison with Other Decision Aids

The proposed decision aid tool aligns with the broader tradition of decision-support interventions developed for complex medical contexts, where multiple therapeutic options are available and uncertainty regarding benefits and risks remains high [18,43]. For example, decision aids used in the treatment of cancer or cardiovascular diseases have demonstrated significant benefits in terms of patient satisfaction and adherence to chosen treatments [22,39,41,44,45]. However, very few tools have been specifically designed to support decision making in palliative care, highlighting the innovative nature of this initiative within the field of existential distress management [9,46].

### 4.2. Strengths and Limitations

The development process had several strengths, including using established frameworks for decision aid design, guidance from a multidisciplinary steering group, incorporation of direct participant feedback, and data collection through semi-structured interviews. The involvement of patient partners throughout the development process reinforces the decision aid’s clinical relevance and validity. However, several limitations must also be acknowledged. The recruitment for this study was challenging, potentially due to the demands of the interview process on participants. Individuals living with serious illnesses often face time and energy constraints, making participation more difficult. In addition, the therapeutic use of psilocybin remains highly innovative, limiting the number of patients and healthcare professionals interested in and qualified to participate. Lastly, there is a scarcity of professionals with experience evaluating decision aids related to such emerging therapies, further reducing the recruitment pool. As a result, the sample size is relatively small (*n* = 10), relatively homogeneous (predominantly highly literate participants from the same geographic area), which limits the generalizability of the findings and warrants caution against broad application at this stage. Nevertheless, data saturation appeared to have been reached during the semi-structured interviews, suggesting that the main relevant themes were adequately explored [47]. A larger-scale study could help confirm these findings and further deepen the understanding of decision-making needs in the context of existential distress [48]. A further limitation concerns the use of a single coder for the qualitative analysis. This approach was supported by using field notes and a reflexive journal to enhance transparency. To help mitigate this limitation, the first author (A.B.) documented the data analysis process and regularly met with the last author (M.D.) to discuss and resolve disagreements, ensuring consensus at each stage [49]. Furthermore, the process was iteratively reviewed by the multidisciplinary steering committee. This group provided oversight, validated emerging themes, and ensured that the analysis remained balanced, rigorous, and reflective of diverse perspectives, thereby helping to reduce the risk of bias inherent in single-coder analysis.

A related consideration is that only a small number of participants re-evaluated the final version of the decision aid, which limited the possibility of a full second round of validation. Nevertheless, the feedback obtained indicated improvements in clarity, balance, and usefulness, and the final version was subsequently reviewed and endorsed by team members with lived experience of PAT.

It is also worth noting that while healthcare professional participants had substantial experience in palliative care, they were all located in the same geographical region, limiting the diversity of perspectives. Most patients living with serious illness participants were also highly educated and health literate, which may have biased the evaluation of the decision aid’s accessibility. To address this, future studies should include participants with broader literacy levels to better assess the decision aid’s usability across diverse populations. Also, most patient participants in this study were women, which may have influenced their informational needs and approaches to values clarification. Achieving greater gender balance in future research could help explore whether men have different perspectives or decision-making preferences in this context.

Lastly, although the decision aid was originally developed in French and translated into English, linguistic and cultural nuances may still affect its interpretation and applicability in other settings. To address this, systematic cultural adaptation of translations, cognitive debriefing with patients and healthcare professionals from diverse backgrounds, and pilot testing in multiple contexts will be required to ensure clarity, accessibility, and relevance across settings.

### 4.3. Clinical Implications

This study underscores the potential role of a decision aid in assisting patients in navigating complex end-of-life choices. The decision aid offers more than just informational content, as is typical of an education booklet. By clarifying the risks and benefits associated with PAT, the decision aid promotes informed decision-making aligned with patients’ values while enhancing dialogue with healthcare professionals. It was designed to support patients in engaging in informed conversations with healthcare professionals, helping them assess the relevance of PAT or consider other options, and present the available options for addressing existential distress in a clear and accessible manner [19].

### 4.4. Research Perspectives

The findings of this study open several avenues for future research. It would be valuable to assess the decision aid’s actual impact on the quality of decision-making and patient experiences over the longer term, particularly by comparing decision trajectories with and without the decision aid. In this regard, conducting a randomized controlled trial would be an essential next step to rigorously evaluate the decision aid’s effectiveness in supporting patient decision-making. Given the growing interest in PAT and the time-intensive nature of conducting a randomized trial, we felt it was important to promptly make the decision aid tool available for clinical use. Feedback is currently being gathered from real-world settings to inform the development of an improved future edition. These efforts are being carried out in collaboration with interdisciplinary teams across multiple institutions and will contribute to future initiatives aimed at scaling up implementation and promoting broader dissemination across diverse care environments.

Developing digital formats, such as mobile applications or interactive platforms, could further improve the decision aid’s accessibility, especially for patients with varying literacy levels. These technologies would also facilitate their integration into clinical workflows by offering real-time access to patient preferences.

Finally, the methodological approach developed here could be extended to other emerging therapies, such as ketamine-assisted therapy or psychospiritual interventions, by designing similar decision support tools tailored to complex medical decision-making contexts. The decision aid will be made available as a downloadable PDF or printed format from the research team’s website: www.p3a.ca.

## 5. Conclusions

This decision aid for PAT represents a clinically relevant and innovative contribution to palliative care, offering structured support for complex decision-making in an area where few educational tools currently exist. It is designed to meet the needs of patients facing existential distress and offers a structured, patient-centered approach grounded in evidence-based practices. By providing balanced and accessible information, the decision aid aims to support patients during critical decision-making moments, fostering choices that are informed and aligned with their personal values.

While its potential for broader use through translation and digital adaptation is promising, these applications remain preliminary. Given the pilot nature of this work and the small, relatively homogeneous sample, caution is warranted before considering broader implementation. Larger and more diverse validation studies will be essential to confirm its acceptability, usability, and impact in real-world settings. Nonetheless, this research highlights the importance of developing holistic and empathetic approaches in end-of-life care while contributing to a broader reflection on integrating emerging therapies into contemporary medicine. It also reinforces the critical role that decision aids can play in improving the patient experience and supporting shared decision making.

## Figures and Tables

**Table 1:** Participant characteristics.

Characteristics	Patients, *n* = 5
**Gender, *n* (%)**	
Male	1 (20)
Female	4 (80)
**Age, *n* (%)**	
35–44 years	1 (20)
45–54 years	1 (20)
55–64 years	2 (40)
65 years and more	1 (20)
**Health conditions, *n* (%)**	
Cancer	3 (60)
Degenerative illness	2 (40)
**Education level, *n* (%)**	
Undergraduate degree	3 (60)
Graduate degree or higher	2 (40)
**Characteristics**	**Healthcare professional, *n* = 5**
**Profession, *n* (%)**	
Family physician	3 (60)
Psychiatrist	2 (40)
**Gender, *n* (%)**	
Male	3 (60)
Female	2 (40)

**Table 2:** Selected quotes—Patients living with serious illness.

Theme	Quotes	Summary of Content
**Balance of** **options**	*“Well, I felt that it leaned more towards proposing psilocybin, but what are the other alternatives really? There aren’t many effective alternatives, and I think that’s a problem.”*	Perceived imbalance favors PAT, but also acknowledges the limited availability of effective alternatives.
**Content**	*“When you said that psilocybin-assisted therapy involves taking a large dose of psilocybin, I thought, oh my god, they’re going to give me a huge dose, I’ll feel terrible and have a bad trip.”*	The term “large dose” was perceived as anxiety inducing; suggestion to replace it with “therapeutic dose” or “high dose.”
**Clarity of** **information**	*“The values clarification table isn’t very clear. Maybe add the actual question directly instead of just the theme, or provide more context.”*	Suggestion to improve the clarity of the tables to facilitate understanding.
**Length**	*“Well, I found the tool maybe a little well, a little long. If it were more visual, I think it would be better.”*	The tool is perceived as somewhat long; adding visual elements and removing less essential information is recommended to enhance accessibility.
**Most appreciated**	*“Overall, I think the tool is really good. It covers all the essential questions.”*	Overall positive appreciation; the tool is deemed as relevant and complete in addressing users’ needs.

**Table 3:** Selected quotes—Healthcare professionals.

Theme	Quotes	Summary of Content
**Balance of options**	*“I found that it pushed a lot, a lot toward psilocybin-assisted therapy rather than toward other treatments. It feels like there’s a bias for psilocybin-assisted therapy, as if PAT is easy and works well, while other treatments are complicated and expensive.”*	Perceived bias in favor of PAT; need to include more information on alternatives such as psychotherapy and ketamine-assisted therapy.
**Content**	*“Sometimes, afterward, you’re completely knocked out for two weeks, you can’t even leave your house, it’s not the be-all and end-all.”*	Warning about the potential for temporary symptom exacerbation following therapy; importance of highlighting possible challenges.
**Clarity of information**	*“I would say, the closer you move to the left side, the more this values clarification seems in favor of PAT.”*	Confusion regarding the values clarification scale; need to simplify and better contextualize this section.
**Length**	*“Everything included in the tool is relevant, there’s nothing superfluous, I think.”*	Tool length generally considered appropriate.
**Most appreciated**	*“I think the heart of the tool is the values. If there’s one thing to simplify, it’s that. All the other information could be given verbally.”*	Values clarification perceived as essential, with suggestions to simplify this section.

**Table 4:** Summary of comments and modifications.

Theme	Comments	Modifications Made
**Context and structure**	Need for better organization of sections to ensure logical progression.	Reorganization of the order of steps: addition of an introductory context, distinction between existential distress, demoralization, and depression.
**Balance of options**	Initial content perceived as too oriented toward psilocybin-assisted therapy.	Inclusion of information about other treatments (psychotherapy, ketamine-assisted therapy, spiritual interventions) to maintain balance.
**Language and literacy**	Language considered too complex by some users.	Simplification of complex terms (“mystical,” “unconscious”); use of accessible and inclusive wording.
**Psilocybin-assisted therapy**	Expectations considered too high and lack of clarity regarding steps and benefits.	Reduction of terms that could inflate expectations, inclusion of medical evaluation steps, simplification of explanations about benefits and risks.
**Risks and contraindications**	Insufficient information provided about certain risks and contraindications.	Addition of specific contraindications (e.g., liver problems, brain injuries) and simplification of the symptom and drug interaction tables.
**Comparative treatment table**	Perceived as too dense and not very visual.	Revision of the table: addition of spiritual interventions, clarification of descriptions, presentation of benefits and risks for each option, inclusion of ketamine therapy.
**Values clarification scale**	Scale perceived as unclear and difficult to use.	Complete revision of the scale, ensuring consistency and providing better contextual information.
**FAQ section**	Need for simplifications and addition of practical information.	Simplification of terminology, addition of advice for consulting a healthcare provider, clarification about the experimental status of the therapy.
**Documentary resources**	Request for easier access to additional information.	Addition of a dedicated section for resources for patients and healthcare professionals.

## Data Availability

Data supporting this study cannot be made available for ethical restrictions as it would compromise participant confidentiality.

## Supplementary Materials

The following supporting information can be downloaded at: https://www.mdpi.com/article/10.3390/healthcare13182290/s1, Supplementary Files S1: SUNDAE Checklist; S2: COREQ Checklist; S3: Reading Grid, S4: Interview Guide, S5: Decision Aid (English version); and S6: Decision Aid (French version). Reference [33] is cited in Supplementary Materials.

## Abbreviations

The following abbreviations are used in this manuscript:

COREQ	Consolidated Criteria for Reporting Qualitative Research
FAQ	Frequently Asked Questions
IPDAS	International Patient Decision Aids Standards Collaboration
PAT	Psilocybin-Assisted Therapy
SUNDAE	Standards for Universal Reporting of Patient Decision Aid Evaluation
