# Supplementary material for: Supporting Meaningful Choices: A Decision Aid for Individuals Facing Existential Distress and Considering Psilocybin-Assisted Therapy
Source: Healthcare (Basel). 2025 Sep 12;13(18):2290. doi: 10.3390/healthcare13182290 (PMC12469295; doi:10.3390/healthcare13182290)
Supplement: Supplementary file 1 [file healthcare-13-02290-s001.zip › Supplementary File S1. SUNDAE_Checklist_Decision Aid_Bélanger et al.pdf]

SUNDAE Checklist Mapping

Bélanger, A., Chang, S.-L., Stephan, J.-F., Moureaux, F., Tapp, D., Foxman, R., Gagnon, P., Hébert, J., Farzin, H., & Dorval, M. (2025). Supporting Meaningful Choices: A Decision Aid for Individuals Facing Existential Distress and Considering Psilocybin-Assisted Therapy.

Item No.	SUNDAE Checklist Item	Corresponding Section in Article
1	Identification as a patient decision aid evaluation study	Title, Abstract
2	Background and objectives	Introduction
3	Need for the decision aid	Introduction
4	Description of the decision aid	Methods – Prototype development, Results – Final version
5	Development process of the decision aid	Methods – Design of the prototype
6	Theoretical framework	Methods – IPDAS, Ottawa Decision Support Framework
7	Format, delivery, and components of the decision aid	Methods – Prototype development
8	Target population	Methods – Sampling and participant recruitment
9	Rationale for targeted population	Methods – Sampling rationale implicitly described
10	Evaluation methodology	Methods – Data Collection, Interviews
11	Setting and context	Methods – Sampling and context (Québec, palliative care)
12	Recruitment methods	Methods – Sampling and participant recruitment
13	Participant characteristics	Results – Participant characteristics, Table 1
14	Comparator (if any)	Not applicable (no comparator used)
15	Evaluation procedure	Methods – Data collection, Semi-structured interviews
16	Data collection instruments	Methods – Reading grid, Interview guide
17	Analysis methods	Methods – Data analysis
18	Main outcome results	Results – Evaluation using the structured assessment grid, Thematic analysis
19	Extent of decision aid use (if measured)	Not applicable
20	Unintended consequences or additional findings	Results – Length and structure, Clarity themes
21	Subgroup findings	Results – Differences between patients and professionals
22	Study limitations	Discussion – Strengths and limitations
23	Implications for practice	Discussion – Clinical implications
24	Suggestions for future research	Discussion – Research perspectives
25	Conflicts of interest	Conflict of interest section
26	Funding	Funding section

Source: Sepucha KR, Abhyankar P, Hoffman AS, et al. Standards for UNiversal reporting of patient Decision Aid Evaluation studies: the development of the SUNDAE Checklist. BMJ Qual Saf. 2018;27(5):389-412. doi:10.1136/bmjqs-2017-006985.
