# Supplementary material for: Supporting Meaningful Choices: A Decision Aid for Individuals Facing Existential Distress and Considering Psilocybin-Assisted Therapy
Source: Healthcare (Basel). 2025 Sep 12;13(18):2290. doi: 10.3390/healthcare13182290 (PMC12469295; doi:10.3390/healthcare13182290)
Supplement: Supplementary file 1 [file healthcare-13-02290-s001.zip › Supplementary File S2. COREQ Checklist_Decision Aid_Bélanger et al. pdf.pdf]

COREQ Checklist – Consolidated Criteria for Reporting Qualitative Research

Bélanger, A., Chang, S.-L., Stephan, J.-F., Moureaux, F., Tapp, D., Foxman, R., Gagnon, P., Hébert, J., Farzin, H., & Dorval, M. (2025). Supporting Meaningful Choices: A Decision Aid for Individuals Facing Existential Distress and Considering Psilocybin-Assisted Therapy.

This 32-item checklist is designed for qualitative studies involving interviews and focus groups.

No.	Item	Description	✓
1	Interviewer/facilitator	Which author(s) conducted the interview or focus group?<br><br>*AB, the first author conducted the interviews.*	Methods p.7
2	Credentials	What were the researcher's credentials? (e.g., PhD, MD)<br><br>*Research team members have experience in conducting qualitative health research.*	Not reported as not required by the journal on the title page
3	Occupation	What was their occupation at the time of the study?<br><br>*Members of the research team included researchers, physicians, nurses and patient partners.*	Methods p. 4
4	Gender	Was the researcher male or female?	Methods p. 6
5	Experience and training	What experience or training did the researcher have?	Methods p.6-7
6	Relationship established	Was a relationship established prior to study commencement?<br>*Only for the purposes of this research. Participants were recruited through professional networks or via institutional mailing lists and social media.*	Methods p.5
7	Participant knowledge of the interviewer	What did the participants know about the researcher?<br><br>*Although healthcare professionals were identified through the authors' professional networks, they were not personally acquainted with AB. The data collection was conducted by AB, who had no prior relationship with the participants. Similarly, individuals living with serious illnesses did not know the interviewer, as they were recruited through advertisements and social media.*	N/A

8	Interviewer characteristics	What characteristics were reported about the interviewer/facilitator?<br><br>*AB is a palliative care nurse*	Methods p. 6
9	Methodological orientation and theory	What methodological orientation was stated to underpin the study?<br><br>*Content analysis*	Methods p.7
10	Sampling	How were participants selected?<br><br>*Purposive sampling*	Methods p.5
11	Method of approach	How were participants approached?<br><br>*Professional networks and institutional mailings lists and social media.*	Methods p.5
12	Sample size	How many participants were in the study?<br><br>*10*	Results p.8
13	Non-participation	How many refused or dropped out? Reasons?<br><br>*All recruited participants completed the interviews; no dropouts occurred.*	Results p.8
14	Setting of data collection	Where was the data collected?<br><br>*Via Teams or Zoom or in person*	Methods p.6-7
15	Presence of non-participants	Was anyone else present besides participants and researchers?<br><br>*No, AB was the only person present.*	Methods p.6-7
16	Description of sample	What are the important characteristics of the sample?<br><br>*Important characteristics include gender, age, education level, employment status, and administrative region.*	Table 1 p.8-9
17	Interview guide	Were questions, prompts, guides provided by the authors? Was it pilot tested?<br><br>*It was reviewed by the steering committee and further refined iteratively as themes emerged during data collection.*	Method p. 7
18	Repeat interviews	Were repeat interviews carried out?<br><br>*None were conducted*	NA
19	Audio/visual recording	Did the study use audio or visual recording?	Method p. 7

		Interviews were audio recorded.	
20	Field notes	Were field notes made during and/or after the interview?<br>*Yes, field notes were made.*	Method<br>p.7
21	Duration	What was the duration of the interviews or focus groups?<br><br>*Interviews lasted 1 hour.*	Methods<br>p. 7
22	Data saturation	Was data saturation discussed?<br><br>*Discussed in the manuscript.*	Discussion<br>p. 14
23	Transcripts returned	Were transcripts returned to participants for comment and/or correction?<br><br>*No, transcripts were not returned to participants.*	NA
24	Number of data coders	How many data coders were involved in the analysis?<br><br>*A single coder was involved, described in the Methods and acknowledged as a limitation in the discussion.*	Methods<br>p.7; p. 14
25	Description of the coding tree	Did authors provide a description of the coding tree?<br><br>*No coding tree was included. However, emerging themes are described.*	NA
26	Derivation of themes	Were themes identified in advance or derived from the data?<br><br>*An inductive approach was followed and themes emerged from the data.*	Methods<br>p.7
27	Software	What software, if any, was used to manage the data?<br><br>*NVivo software.*	Methods<br>p.7
28	Participant checking	Did participants provide feedback on the findings?<br><br>*Results were not shared with participants at this stage*	NA
29	Quotations presented	Were participant quotations presented to illustrate the findings?<br><br>*Yes.*	Results<br>Tables 2 and 3

30	Data and findings consistent	Was there consistency between the data and the findings?<br><br>*Yes, similar themes emerged among both groups of participants.*	Results p.10-12
31	Clarity of major themes	Were major themes clearly presented?<br><br>*Five major themes were presented and described.*	Results Tables 2 and 3; p.10-12
32	Clarity of minor themes	Is there a description of diverse cases or minor themes?<br><br>*No, there was no description of diverse cases or minor themes.*	NA
