# Supplementary material for: Supporting Meaningful Choices: A Decision Aid for Individuals Facing Existential Distress and Considering Psilocybin-Assisted Therapy
Source: Healthcare (Basel). 2025 Sep 12;13(18):2290. doi: 10.3390/healthcare13182290 (PMC12469295; doi:10.3390/healthcare13182290)
Supplement: Supplementary file 1 [file healthcare-13-02290-s001.zip › Supplementary File S3. Reading grid_Bélanger et al.pdf]

Supplementary material

Bélanger, A., Chang, S.-L., Stephan, J.-F., Moureaux, F., Tapp, D., Foxman, R., Gagnon, P., Hébert, J., Farzin, H., & Dorval, M. (2025). Supporting Meaningful Choices: A Decision Aid for Individuals Facing Existential Distress and Considering Psilocybin-Assisted Therapy.

<u>Reading grid</u>

Reading Grid

Evaluation of a Decision Aid for Individuals Considering Psilocybin-Assisted Therapy for Existential Distress

Assessment of the Acceptability and Usability of the Decision Aid

1. In your opinion, how satisfactory are the following elements?

Reading Grid	Not at all satisfactory	Somewhat satisfactory	Satisfactory	Very satisfactory	Your comments
Overall, the quality of the booklet:					
**Please evaluate each section of the tool to indicate your thoughts on how the information was presented.**					
**Section 1**: Expand your knowledge about psilocybin-assisted therapy in relieving existential distress.					
**Section 2:** Compare psilocybin-assisted therapy with other available treatment options.					
**Section 3**: Reflect on what matters most to you.					
**Section 4:** Are you ready to make your choice?					

2. The length of the tool presentation is :

- ☐ Too long
- ☐ Too short
- ☐ Appropriate

3. The amount of information in the tool is :

- ☐ Too much information
- ☐ Not enough information

☐ Sufficient information

4. The comprehensibility of the tool's content is:
☐ Difficult to understand
☐ Easy to understand
☐ Comments :

5. I found the presentation of the tool to be:
☐ Oriented more towards managing existential distress through psilocybin-assisted therapy
☐ Oriented more towards managing existential distress through other available options
☐ Well balanced

6. Do you find this decision aid useful for making a decision about psilocybin-assisted therapy?
☐ Yes
☐ No
☐ Comments :

7. Do you think we included enough information to help individuals choose a treatment for existential distress?
☐ Yes
☐ No
☐ Comments :

8. What do you like about the decision aid for individuals considering psilocybin-assisted therapy for existential distress?

____
____
____
____

9. What are your suggestions for improving the decision aid ?

____
____
____
____

References :

Reading grid inspired by O'Connor AM & Cranney A. User Manual – Acceptability. Ottawa: Ottawa Hospital Research Institute, 1996. 5p. Available from http://decisionaid.ohri.ca/docs/develop/User_Manuals/UM_Acceptability.pdf

Santerre-Theil, A., Bouchard, K., St-Pierre, D., Drolet, A. M., Chiquette, J., & Dorval, M. (2018). Development of a Tool to Guide Parents Carrying a BRCA1/2 Mutation Share Genetic Results with Underage Children. *J Cancer Educ*, *33*(3), 569-575. https://doi.org/10.1007/s13187-016-1127-x
