# Supplementary material for: Supporting Meaningful Choices: A Decision Aid for Individuals Facing Existential Distress and Considering Psilocybin-Assisted Therapy
Source: Healthcare (Basel). 2025 Sep 12;13(18):2290. doi: 10.3390/healthcare13182290 (PMC12469295; doi:10.3390/healthcare13182290)
Supplement: Supplementary file 1 [file healthcare-13-02290-s001.zip › Supplementary File S4. Interview guide_Bélanger et al.pdf]

Supplementary material

Bélanger, A., Chang, S.-L., Stephan, J.-F., Moureaux, F., Tapp, D., Foxman, R., Gagnon, P., Hébert, J., Farzin, H., & Dorval, M. (2025). Supporting Meaningful Choices: A Decision Aid for Individuals Facing Existential Distress and Considering Psilocybin-Assisted Therapy.

Interview Guide

The objective of this interview is to explore in depth your impressions of the decision aid. Your detailed responses are essential for refining this tool. Thank you for taking the time to share your thoughts and experiences regarding the following aspects:

Overall, what do you think of the decision aid?

- Could you elaborate on what particularly caught your attention or what could be improved?

What do you think of the title?

- How does the title influence your perception of the tool? Do you have any suggestions to make it more impactful or informative?

What do you think about the order of the tool's steps?

- Should some information be introduced earlier in the document? Are any sections unnecessary?
- Could you provide specific examples of elements that could be reorganized or removed?

What do you think of the content of the decision aid?

- Is there enough information about the available options? Are the options well-balanced?
- Could you provide examples of information that you consider crucial and those that could be expanded? How do you assess the balance of the presented options?

What do you think of the clarity of the information?

- Did you have difficulty understanding certain parts?
- Could you specify the sections where you encountered comprehension difficulties? Do you have any suggestions for making the information more accessible?

Do you think the document includes enough information and tools to help a person choose a treatment for existential distress?

- What additional information or tools could strengthen the tool's effectiveness in decision making?

What did you like most about the decision aid?

- Could you identify specific aspects of the tool that particularly stood out or seemed especially useful?

What suggestions would you make to improve the decision aid?

- Would it be possible to obtain concrete recommendations for specific improvements or additional features?

Do you think this tool helps prepare you to discuss what is important for your decision making with your healthcare professional?

- How could the tool be enhanced to facilitate a more in-depth discussion with healthcare professionals?

Are there any other topics you would like to address regarding this tool that have not been discussed?

- Are there aspects not covered by the previous questions that deserve further exploration? Any specific topics you would like to highlight?

We sincerely appreciate you taking the time to share your detailed perspectives. Your feedback plays a crucial role in the ongoing development of this decision aid.
