# Supplementary material for: Supporting Meaningful Choices: A Decision Aid for Individuals Facing Existential Distress and Considering Psilocybin-Assisted Therapy
Source: Healthcare (Basel). 2025 Sep 12;13(18):2290. doi: 10.3390/healthcare13182290 (PMC12469295; doi:10.3390/healthcare13182290)
Supplement: Supplementary file 1 [file healthcare-13-02290-s001.zip › Supplementary File S5. Decision Aid_English_Bélanger et al..pdf]

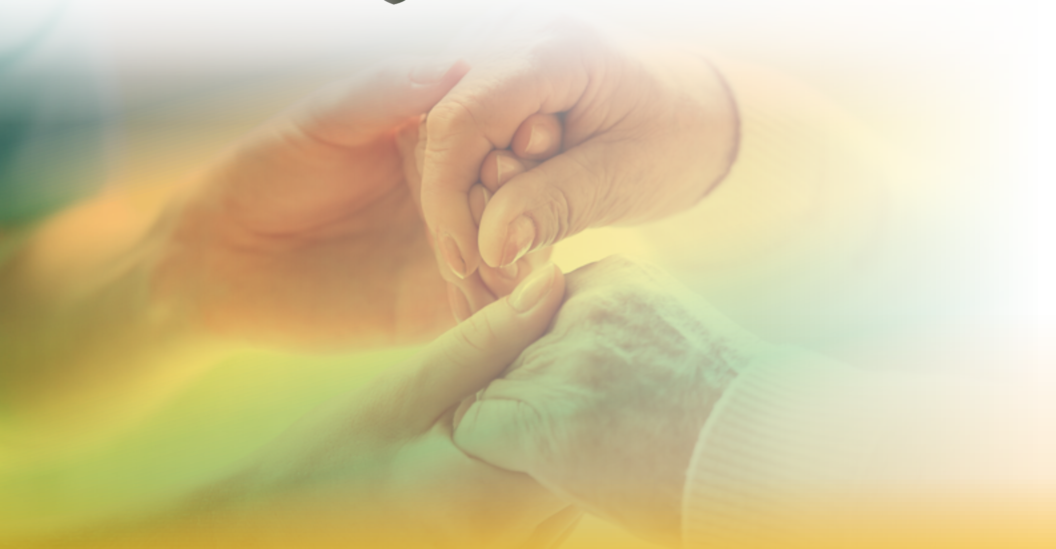

PSILOCYBIN-ASSISTED THERAPY for EXISTENTIAL DISTRESS : Making the right choice for you

A Decision Aid

This tool is for you if :

- You are feeling hopeless due to a serious illness
- You have tried multiple treatments to alleviate this distress
- You want to learn more about available treatments to relieve this distress
- You want to learn more about psilocybin-assisted therapy

This decision aid booklet was created by Ariane Bélanger, under the supervision of Professor Michel Dorval, as part of her Master's in Pharmaceutical Sciences at Université Laval, Quebec, Canada.

EDITORIAL TEAM :

- Ariane Bélanger, BScN.
- Michel Dorval, Ph.D.
- Sue-Ling Chang, M.Sc.
- Florence Moureaux, patient partner
- Robert Foxman, patient partner
- Jean-François Stephan, M.D.
- Houman Farzin, M.D.
- Diane Tapp, Ph.D.

FINANCIAL PARTNERS :

- Équipe de Recherche Michel-Sarrazin en Oncologie psychosociale et Soins palliatifs (ERMOS)
- Chaire de recherche en soins palliatifs de l'Université Laval
- Fonds d'enseignement et recherche (FER) de la Faculté de pharmacie de l'Université Laval

We warmly thank all those who contributed to the development of this booklet by providing feedback on previous versions. These contributions were gathered as part of a research project approved by the Research Ethics Committee of CHU de Québec–Université Laval (#2024-7116).

Created by Ariane Bélanger, Michel Dorval, Sue-Ling Chang
© Université Laval 2025. All rights reserved.

No part of this document may be modified, and no element may be used out of context without written permission. For additional information, please contact p3a@crchudequebec.ulaval.ca

Please cite this document as follows: *Bélanger A, Dorval M, Chang SL. (2025) Psilocybin-assisted therapy for existential distress: Making the right choice for you. A decision aid. Université Laval, Québec.*

P3A
Psilocybine en fin de vie
Audace, Acceptabilité, Accès

UNIVERSITÉ LAVAL

Is *PSILOCYBIN*-assisted THERAPY right for you?

To alleviate existential distress

Learn more about existential distress and the treatments that may help 5

1.1 What is existential distress ? 5

1.2 What are the treatments for existential distress ? 6

Learn more about psilocybin-assisted therapy 8

2.1 What is psilocybin ? 8

2.2 What is psilocybin-assisted therapy ? 9

2.3 What are the benefits ? 12

2.4 What are the risks ? 13

2.5 What are the contraindications ? 15

2.6 What are the drug interactions ? 16

Think about what matters most 17

Are you prepared to make your decision? 20

Resources 24

Bibliography 26

LEARN MORE about existential distress and the treatments that may help

1.1 | What is existential distress?

Being diagnosed with a serious illness can affect your emotions, relationships, and spiritual life, leading to existential distress. About one-third of people with advanced cancer or another serious illness experience feelings of existential distress, anxiety, and depression.

Symptoms include :

- Feelings of discouragement, hopelessness, loss of meaning, and helplessness
- A loss of meaning in life
- The fear of dying or the desire to die
- Symptoms of depression and anxiety

> ***It is important to talk about your symptoms with a healthcare professional, as it can be difficult to distinguish a temporary depressive episode from existential distress. Healthcare professionals can help you understand your symptoms and find the best treatment.***

› TABLE OF CONTENTS

1.2 | What are the treatments for existential distress

There are different treatments to help with existential distress. Here is an overview of the options available.

Conventional treatments :

- **Medications :** Some medications, like anxiolytics and antidepressants, may be prescribed to help with the anxiety and depression that often come with existential distress. These medications should be prescribed and monitored by a qualified healthcare professional.
- **Psychotherapy :** Certain types of psychotherapy, like cognitive-behavioral therapy and existential therapy, can help people explore their feelings and find meaning in life despite illness.
- **Spiritual support :** Talking with a spiritual or psychosocial counselor can help people find comfort while considering their values and beliefs in the face of illness.

Psychedelic-assisted therapies :

- **Psilocybin-assisted therapy :** Psilocybin, a substance found in certain mushrooms, coupled with psychotherapy, can help people who are struggling with existential distress. This approach will be explained in more detail in this tool.
- **Ketamine-assisted therapy :** Ketamine, a drug used for anesthesia, is also being used for its potential to help people at the end of life. It has been shown to be effective in treating depression, which is often linked to existential distress.

Complementary treatments :

- **Meditation and yoga :** Approaches like meditation and yoga can help reduce stress and improve overall well-being.

These treatments can be used alone or combined, depending on each person's needs and preferences. It is important to talk to a qualified healthcare professional to figure out the best options for your situation.

In the next section, we will take a closer look at psilocybin-assisted therapy to help you decide if this approach could be an option to ease your existential distress.

› TABLE OF CONTENTS

LEARN MORE about psilocybin-assisted therapy

In this section, we will cover what you need to know about psilocybin, psilocybin-assisted therapy, its benefits, risks, contraindications, and possible drug interactions.

2.1 | What is psilocybin

Psilocybin is a natural substance found in certain mushrooms, often referred as “magic mushrooms.” Once in the body, it is turned into a substance that affects the brain. It acts on a molecule called serotonin, which plays an important role in managing emotions and mood.

When used in a therapeutic setting, psilocybin can temporarily change how the brain works, making different parts of the brain more connected. This can help people see things differently or explore new ways of understanding their emotions.

Some people report feeling calmer or finding meaning after their psilocybin experience. However, these effects can vary greatly from person to person, and there is no guarantee that psilocybin will have these benefits for everyone.

Psilocybin-assisted therapy is still being studied and is only used in specific cases. In Canada, it is available through **Health Canada’s Special Access Program** for patients with serious illnesses. It can be accessed when other treatments have not worked or are not suitable.

In the next section, we will explain how psilocybin-assisted therapy works.

2.2 | What is psilocybin-assisted therapy ?

Before starting psilocybin-assisted therapy, a medical evaluation is required to make sure the therapy is safe for you.

Psilocybin-assisted therapy involves taking a moderate dose of psilocybin while undergoing psychotherapy. It happens in three important steps: 1) preparation, 2) psilocybin administration, and 3) integration. These steps are essential for the success of the therapy and affect its results.

1 | The first step is the **preparation**

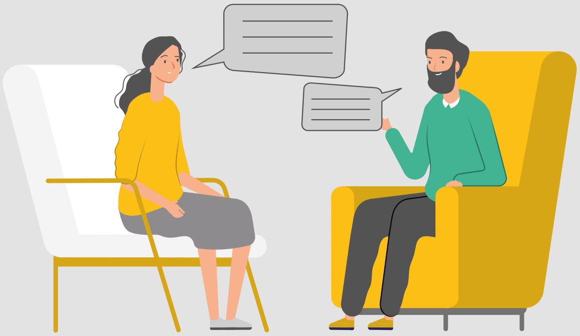

This step is about building a trusting relationship with a healthcare professional who will be your therapist and guide you during the psilocybin administration session. Your existential distress will also be explored in different ways to define the goal of the treatment. The preparatory meetings are meant to understand your expectations and prepare you for the treatment, ensuring it is done safely by providing you with the necessary information for the next step.

› TABLE OF CONTENTS

2 | The second step is the **psilocybin administration session**

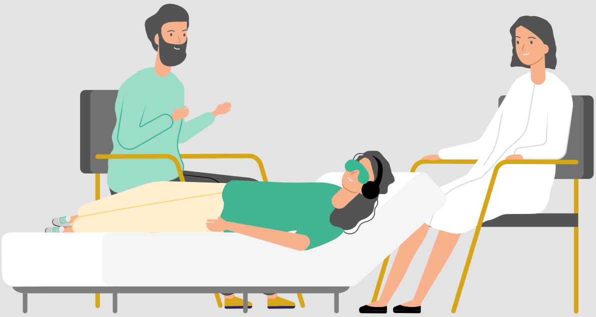

The session normally takes place in a comfortable and calming space made for therapy. Sometimes, it can be in your home. You will have the option to lie down with an eye mask and headphones to eliminate distractions and deepen your experience. Two healthcare professionals will accompany you during the session and typically one of them will be your prescribing doctor. The session lasts about 6-8 hours and includes discussions with your therapists who are there to support you and discuss your experience.

3 | The third step is the **integration**

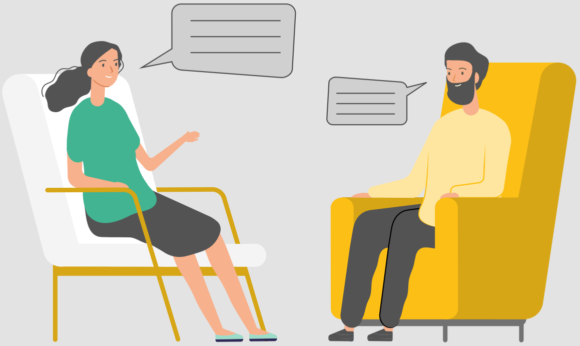

This step involves talking with your therapist about what you experienced during the psilocybin session. It is similar to regular psychotherapy and can take place in one or more sessions. The main goal is to help you make sense of what you went through during the psilocybin session. The integration process also helps you apply these changes to your everyday life. For some people, this may take several months.

2.3 | What are the possible benefits of psilocybin-assisted therapy ?

Recent studies show that taking psilocybin just once, coupled with psychotherapy, can have the following effects :

- A fast and strong decrease in symptoms of depression and anxiety.
- An improvement in feelings of sadness, hopelessness, or loss of purpose.
- Less fear of dying and better acceptance of death.
- Better quality of life and improved mental and spiritual well-being.

> ***According to some studies, these effects can last for at least 6 months after just one dose of psilocybin.***

2.4 | What are the possible risks of psilocybin-assisted therapy?

Physical effects	Psychological effects
**Most common side-effects (>5%)**	
Increase in blood pressure<br>Increase in heart rate<br>Headache<br>Nausea (feeling like vomiting)	Anxiety (feeling very worried)<br>Confusion (having trouble understanding what's going on)
**Less common side-effects (<5%)**	
Fatigue<br>Migraine (severe headaches)<br>Vomiting<br>Physical discomfort (feeling unwell in your body)	Difficult experiences (also called *bad trip*)<br>Psychological discomfort (feeling mentally unwell)<br>Spiritual crisis (questioning important values)<br>Psychiatric complications (worsening of certain mental health issues)

Based on MacCallum et al.,2022, *Frontiers in Psychiatry.*

These potential side-effects are known from studies done in a controlled setting (with a lot of supervision). In less supervised situations, these side-effects could be more common.

Sometimes, psilocybin can lead to uncomfortable feelings, like anxiety or feeling very uneasy. These experiences, known as "bad trips," usually do not last long. Some people may feel let down if the therapy does not give them the results they hoped for, which could make their original problem worse.

People with a personal or family history of mental health problems should discuss this with their healthcare professional before starting this type of therapy. Although rare, there is a risk of mental health complications, like psychotic symptoms.

Preparation sessions are very important to reduce these risks. They help people understand the process and get ready for any difficult

experiences. During the therapy, professionals provide constant supervision, making the treatment as safe as possible.

Even though an experience might be difficult at the time, it can be helpful with the integration work done during psychotherapy. This follow-up helps people get the most out of their experience.

2.5 | What are the contraindications?

There are no strict contraindications for psilocybin-assisted therapy. This means that, for most people, it is possible to talk to a doctor to decide if it is a good option for them. The main reasons not to use this therapy are related to certain health problems. For example:

- **Serious or uncontrolled heart problems :** Such as heart failure, uncontrolled high blood pressure, or severe arrhythmia.
- **Liver failure:** Specific issues with the liver.
- **Certain brain conditions:** Specific issues with the brain.
- **A history of psychosis:** For example, disorders like schizophrenia and bipolar disorder.
- It is also not recommended to use this therapy during **pregnancy** and **breastfeeding** because there is not enough scientific evidence about the possible risks.

> ***To see if this therapy is right for you, it is important to discuss the risks and benefits with your doctor. This decision is made together with your doctor, who will assess your health and help you make the best choice for you.***

2.6 | What are the possible drug interactions?

Before starting psilocybin-assisted therapy, it is important to talk to a healthcare professional about the medications you are already taking. Some medications can interact with psilocybin, and your doctor can advise you on any needed adjustments.

For cancer patients going through chemotherapy and radiation treatments, current studies show no evidence of negative interactions with psilocybin. In clinical studies, these treatments have continued without noticeable problems related to psilocybin, but it is still important to talk to your doctor.

> ***Talk to your doctor to understand the potential risks of drug interactions with psilocybin-assisted therapy.***

THINK ABOUT
what matters most

Use the scale on the following pages to help evaluate your personal values regarding psilocybin-assisted therapy. On this scale, the more your evaluation is on the left side, the more you favour psilocybin-assisted therapy. For example, if you strongly value avoiding difficult emotions, it might affect your position on the scale. Discuss your answers and thoughts with your healthcare professional to make an informed decision.

1 | Quality of life

The symptoms affect my quality of life and my daily activities	❑ Completely applies to me	❑ Somewhat applies to me	❑ I do not know	❑ Somewhat applies to me	❑ Completely applies to me	I am able to function and my quality of life is acceptable

2 | Fear of death

I am afraid of death and it causes me distress	❑ Completely applies to me	❑ Somewhat applies to me	❑ I do not know	❑ Somewhat applies to me	❑ Completely applies to me	I am not afraid of death, and anxiety does not cause me distress

3 | Other treatments

I tried other treatments, but they did not relieve my symptoms	❑ Completely applies to me	❑ Somewhat applies to me	❑ I do not know	❑ Somewhat applies to me	❑ Completely applies to me	I have not tried other treatments to relieve my symptoms

4 | Ability to handle emotions

I am ready to experience emotions that might be difficult as part of therapy	❑ Completely applies to me	❑ Somewhat applies to me	❑ I do not know	❑ Somewhat applies to me	❑ Completely applies to me	I am unable to handle any more difficult emotions, and I need immediate relief

› TABLE OF CONTENTS

5 | Side-effects

My symptoms are worse than the possible side-effects of psilocybin	❑ Completely applies to me	❑ Somewhat applies to me	❑ I do not know	❑ Somewhat applies to me	❑ Completely applies to me	I believe the side-effects will be harder to handle than my current symptoms

6 | Commitment to therapy

I am ready to take the necessary steps to get access to therapy	❑ Completely applies to me	❑ Somewhat applies to me	❑ I do not know	❑ Somewhat applies to me	❑ Completely applies to me	I am not ready to take the necessary steps, as it requires too much time

7 | Openness to spirituality

I am open to developing my spirituality and letting myself be guided by an introspective experience	❑ Completely applies to me	❑ Somewhat applies to me	❑ I do not know	❑ Somewhat applies to me	❑ Completely applies to me	I am not open to spiritual development, and I do not feel capable of being guided by an introspective experience

8 | Intention to start therapy

I am ready to commit to therapy with multiple ses-sions that requires time and self-reflection	❑ Completely applies to me	❑ Somewhat applies to me	❑ I do not know	❑ Somewhat applies to me	❑ Completely applies to me	I need immediate relief, and I do not want to get involved in therapy

Are you prepared to make your DECISION ?

Frequently Asked Questions (FAQ) :

Is psilocybin-assisted therapy for everyone ?

For some people, psilocybin-assisted therapy might not work as expected, sometimes leading to disappointment or even hopelessness. This therapy requires a lot of effort from the patient. It is important to talk openly with your doctor about your expectations and goals for this treatment.

What can I expect from psilocybin-assisted therapy ?

The treatment helps explore thoughts and feelings that are hard to express normally. You might notice changes in how you see things. The effects can be different for each person. Studies show that many people feel less anxiety and depression after the treatment, but it does not work for everyone.

Is there a risk of addiction ?

Psilocybin does not cause physical dependence and has a low level of toxicity. It is considered safe when used under medical supervision.

What is the cost of the treatment ?

Currently in Quebec, psilocybin is supplied free of charge and the Régie de l'assurance maladie du Québec (RAMQ) usually covers the cost of treatment when given by doctors or through a hospital system. When the treatment is done outside of these contexts, usually the doctor's fees will be covered but there will be costs for other healthcare professionals involved.

How to get access to the treatment ?

You can get access to the treatment through **Health Canada's Special Access Program**. A doctor has to apply for it. You should talk to your doctor to see if they are willing to help you with this process. If not, they might be able to send you to someone who can give you more information. If needed, you can also reach out to TheraPsil. It is a non-profit group that helps Canadians who

qualify for psilocybin-assisted therapy. But even if your doctor applies through the Special Access Program, there's no guarantee it will be approved. It is important to note that this is an exceptional treatment.

What is microdosing? Is it an alternative ?

Microdosing with psilocybin involves taking about 1/10 to 1/20 of the usual therapeutic dose almost every day to help with anxiety and depression. Even though more and more Canadians are trying it, psilocybin is still illegal, and studies on microdosing do not give clear answers. No current evidence supports this method, so it is not recommended.

Upon reflection, I have reached the following conclusion:

- ❑ I am certain that I want to consider psilocybin-assisted therapy.
- ❑ I think other treatments might be more appropriate for me :
  - Medications
  - Psychotherapy
  - Spiritual support
  - Complementary therapies (meditation, yoga)
- ❑ I am not certain which treatment would be best for me, but I am leaning towards the following option:

› TABLE OF CONTENTS

> ***Talk to your doctor to see if combining different treatments could give you better overall support.***

Notes and reflections:

If you are uncertain whether psilocybin-assisted therapy is right for you, the following chart may help you identify the aspects that are making your decision hard. You can highlight what applies to you and find ways to address your needs.

What makes it hard for me to decide	Actions to consider
I do not have enough information about other treatments for existential distress and their risks and benefits.	Make a list of my questions<br>Look for reliable resources I can use to find the information I need (e.g., healthcare professionals, scientific studies, clinical trials, the internet, non-profit organizations, etc.).
I am not sure if I am dealing with existential distress, anxiety, or depression.	Talk about my symptoms with a healthcare professional.
I am not sure whether the benefits or the downsides of psilocybin-assisted therapy are more important to me.	Talk to a healthcare professional to understand how other people with similar symptoms made their decision.<br>Read testimonials from other people who chose psilocybin-assisted therapy.
I lack the support and resources to make an informed decision.	Get the advice of a healthcare professional and talk with someone I trust.

Share your answers with your doctor.

RESOURCES

Resources for healthcare professionals (Web Resources)

Resource	Description
TheraPsil	A Canadian non-profit organization that helps healthcare professionals navigate the process of accessing psilocybin-assisted therapy. therapsil.ca
Multidisciplinary Association for Psychedelic Studies (MAPS)	A research organization providing information and resources on psychedelics in therapy. maps.org
Psychedelic Medicine Podcast – Dr. Lynn Morski	Several podcasts are dedicated to discussing the therapeutic uses of psychedelics, including psilocybin, with experts in the medical field. plantmedicine.org
The American Trip – Ido Hartogsohn	A book that explores the concept of “set and setting” and its impact on psychedelic experiences,
Découverte television report which aired on November 12, 2023	A Découverte report that follows a patient through the process of psilocybin-assisted therapy. ici.radio-canada.ca/tele/decouverte/site/episodes/836954/psilocybine-therapie-psychedelique-sante-mentale-depression (in French)

Publications and Studies

1 | **Griffiths, R. R., et al. (2016)** : *Psilocybin produces substantial and sustained decreases in depression and anxiety in patients with life-threatening cancer: A randomized double- blind trial. Journal of Psychopharmacology, 30(12), 1181-1197. PubMed.*

2 | **Ross, S., et al. (2016)** : *Rapid and sustained symptom reduction following psilocybin treatment for anxiety and depression in patients with life-threatening cancer: A randomized controlled trial. Journal of Psychopharmacology, 30(12), 1165-1180. PubMed.*

3 | **Grob, C. S., et al. (2011)** : *Pilot study of psilocybin treatment for anxiety in patients with advanced-stage cancer. Archives of General Psychiatry, 68(1), 71-78. PubMed.*

4 | **MacCallum, C. A., et al. (2022)** : *Therapeutic use of psilocybin: Practical considerations for dosing and administration. Frontiers in Psychiatry, 13, 1040217. PubMed.*

5 | **Nichols, D.E. (2016)** : *Psychedelics. Pharmacological Reviews, 68(2), 264-355. PubMed.*

6 | **National Cancer Institute. (2023)** : *Ongoing Clinical Trials on Psilocybin in Palliative Care. National Cancer Institute.*

7 | **Griffiths, R. R., et al. (2023)** : *Long-term Follow-up of Psilocybin- Assisted Psychotherapy for Psychiatric and Existential Distress in Patients with Life-threatening Cancer. Journal of Psychopharmacology. PubMed.*

Resources for the public
(Web Resources)

Resource	Description
TheraPsil	Information and support for Canadian patients seeking access to psilocybin-assisted therapy. therapsil.ca
Psychedelic Support	A platform providing information and support for people exploring psychedelic treatments, including psilocybin. psychedelic.support/resources/psilocybin-assisted-therapy-guide

Documentaries

1 | **Radio-Canada - Au cœur d'une thérapie psychédélique** : A Radio-Canada report that explores psilocybin-assisted therapy. ici.radio-canada.ca/info/long-format/2025449/coeur-therapie-psychedelique-psilocybine (in French)

2 | **Dosed : The trip of a lifetime – Golden Teacher film** : A documentary that explores the therapeutic effects of psilocybin. dosedmovie.com

Podcasts

1 | **Radio-Canada – Voyage inédit au cœur des thérapies assistées par les psychédéliques :** A Radio-Canada podcast that explores the medical use of psychedelics. ici.radio-canada.ca/ohdio/balados/6108/ca-sexplique-balado-info-alexis-delancer/837309/drogue-therapie-champignons-lsd-psychedeliques-experience (in French)

2 | **France Culture - Explorations psychédéliques :** A series of podcasts that explore the medicinal uses of psychedelics, including psilocybin. radiofrance.fr/france-culture/podcasts/serie-explorations-psychedeliques (in French)

3 | **Plant Medicine Podcast** : Podcasts that discuss psychoactive substances with experts, including psychedelics like psilocybin, and their therapeutic uses. The content is more in-depth and detailed, but still accessible to those with a strong interest in psychedelics. plantmedicine.org

4 | **Huberman Lab - How Psilocybin Can Rewire Our Brain, Its therapeutic Benefits & its Risks :** A podcast episode that explores how psilocybin can rewire our brain, its therapeutic benefits, and its risks. www.hubermanlab.com/episode/how-psilocybin-can-rewire-our-brain-its-therapeutic-benefits-and-its-risks

5 | **Numinus - Psychedelic Therapy Frontiers :** How to prepare for, navigate, and integrate a psychedelic experience.youtube.com/@numinusnetwork

Livres

1 | **How to Change your Mind– Michael Pollan (2018)** : This book explores the therapeutic use of psychedelics, including psilocybin, and their effects on mental health.

2| **The American Trip - Ido Hartosohn** : A book about the importance of 'set and setting' in psychedelic experiences.

3| **Phantastica: ces substances interdites qui guérissent - Stéphanie Chayet (2020)** : Based on the author's experience, this book questions the distinction between drugs and medicine and shows the effects of using psychedelics (in French).

BIBLIOGRAPHY

Breitbart, William, and Harvey Chochinov. *Handbook of Psychiatry in Palliative Medicine 3rd Edition*. 2022. doi:10.1093/med/9780197583838.001.0001.

Chochinov, H. M., L. J. Kristjanson, W. Breitbart, S. McClement, T. F. Hack, T. Hassard, and M. Harlos. "Effect of Dignity Therapy on Distress and End-of-Life Experience in Terminally Ill Patients: A Randomised Controlled Trial." *Lancet Oncol* 12, no. 8 (Aug 2011): 753-62. https://doi.org/10.1016/S1470-2045(11)70153-X. https://www.ncbi.nlm.nih.gov/pubmed/21741309

Clarke, D. M., and D. W. Kissane. "Demoralization: Its Phenomenology and Importance." [In eng]. *Aust N Z J Psychiatry* 36, no. 6 (Dec 2002): 733-42. https://doi.org/10.1046/j.1440-1614.2002.01086.x

Davis, A. K., F. S. Barrett, D. G. May, M. P. Cosimano, N. D. Sepeda, M. W. Johnson, P. H. Finan, and R. R. Griffiths. "Effects of Psilocybin-Assisted Therapy on Major Depressive Disorder: A Randomized Clinical Trial." *JAMA Psychiatry* 78, no. 5 (May 1 2021): 481-89. https://doi.org/10.1001/jamapsychiatry.2020.3285. https://www.ncbi.nlm.nih.gov/pubmed/33146667

Griffiths, R. R., M. W. Johnson, M. A. Carducci, A. Umbricht, W. A. Richards, B. D. Richards, M. P. Cosimano, and M. A. Klinedinst. "Psilocybin Produces Substantial and Sustained Decreases in Depression and Anxiety in Patients with Life-Threatening Cancer: A Randomized Double-Blind Trial." *J Psychopharmacol* 30, no. 12 (Dec 2016): 1181-97. https://doi.org/10.1177/0269881116675513 - https://www.ncbi.nlm.nih.gov/pubmed/27909165

Grob, C. S., A. L. Danforth, G. S. Chopra, M. Hagerty, C. R. McKay, A. L. Halberstadt, and G. R. Greer. "Pilot Study of Psilocybin Treatment for Anxiety in Patients with Advanced-Stage Cancer." *Arch Gen Psychiatry* 68, no. 1 (Jan 2011): 71-8. https://doi.org/10.1001/archgenpsychiatry.2010.116. https://www.ncbi.nlm.nih.gov/pubmed/20819978

Johnson, M. W., W. A. Richards, and R. R. Griffiths. "Human Hallucinogen Research: Guidelines for Safety." *Journal of Psychopharmacology* 22 (6) (2008): 603-20. https://journals.sagepub.com/doi/10.1177/0269881108093587

Kissane, D. W. , D. M. Clarke, and A.F Street. "Demoralization Syndrome-a Relevant Psychiatric Diagnosis for Palliative Care." [In eng]. *Journal of Palliative Care* 17, no. 1 (2001): 12-21.

Lee, W., C. Sheehan, R. Chye, S. Chang, A. Bayes, C. Loo, B. Draper, M. R. Agar, and D. C. Currow. "Subcutaneous Ketamine Infusion in Palliative Patients for Major Depressive Disorder (Skipmdd)-Phase Ii Single-Arm Open-Label Feasibility Study." *PLoS One* 18, no. 11 (2023): e0290876. https://doi.org/10.1371/journal.pone.0290876 - https://www.ncbi.nlm.nih.gov/pubmed/37963146

LeMay, K., and K. G. Wilson. "Treatment of Existential Distress in Life Threatening Illness: A Review of Manualized Interventions." *Clin Psychol Rev* 28, no. 3 (Mar 2008): 472-93. https://doi.org/10.1016/j.cpr.2007.07.013. https://www.ncbi.nlm.nih.gov/pubmed/17804130

MacCallum, C. A., L. A. Lo, C. A. Pistawka, and J. K. Deol. "Therapeutic Use of Psilocybin: Practical Considerations for Dosing and Administration." *Front Psychiatry* 13 (2022): 1040217. https://doi.org/10.3389/fpsyt.2022.1040217. https://www.ncbi.nlm.nih.gov/pubmed/36532184

Mitchell, A. J., M. Chan, H. Bhatti, M. Halton, L. Grassi, C. Johansen, and N. Meader. "Prevalence of Depression, Anxiety, and Adjustment Disorder in Oncological, Haematological, and Palliative-Care Settings: A Meta-Analysis of 94 Interview-Based Studies." *Lancet Oncol* 12, no. 2 (Feb 2011): 160-74. https://doi.org/10.1016/S1470-2045(11)70002-X - https://www.ncbi.nlm.nih.gov/pubmed/21251875

Oyetunji, A., C. Huelga, K. Bunte, R. Tao, and V. Bellman. "Use of Ketamine for Depression and Suicidality in Cancer and Terminal Patients: Review of Current Data." *AIMS Public Health* 10, no. 3 (2023): 610-26. https://doi.org/10.3934/publichealth.2023043 - https://www.ncbi.nlm.nih.gov/pubmed/37842268

Ross, S., A. Bossis, J. Guss, G. Agin-Liebes, T. Malone, B. Cohen, S. E. Mennenga, *et al.* "Rapid and Sustained Symptom Reduction Following Psilocybin Treatment for Anxiety and Depression in Patients with Life-Threatening Cancer: A Randomized Controlled Trial." *J Psychopharmacol* 30, no. 12 (Dec 2016): 1165-80. https://doi.org/10.1177/0269881116675512 - https://www.ncbi.nlm.nih.gov/pubmed/27909164

Vehling, S., D. W. Kissane, C. Lo, H. Glaesmer, T. J. Hartung, G. Rodin, and A. Mehnert. "The Association of Demoralization with Mental Disorders and Suicidal Ideation in Patients with Cancer." *Cancer* 123, no. 17 (Sep 1 2017): 3394-401. https://doi.org/10.1002/cncr.30749 - https://www.ncbi.nlm.nih.gov/pubmed/28472548

Vehling, S., Y. Tian, C. Malfitano, J. Shnall, S. Watt, A. Mehnert, A. Rydall, *et al.* "Attachment Security and Existential Distress among Patients with Advanced Cancer." *J Psychosom Res* 116 (Jan 2019): 93-99. https://doi.org/10.1016/j.jpsychores.2018.11.018 - https://www.ncbi.nlm.nih.gov/pubmed/30655000

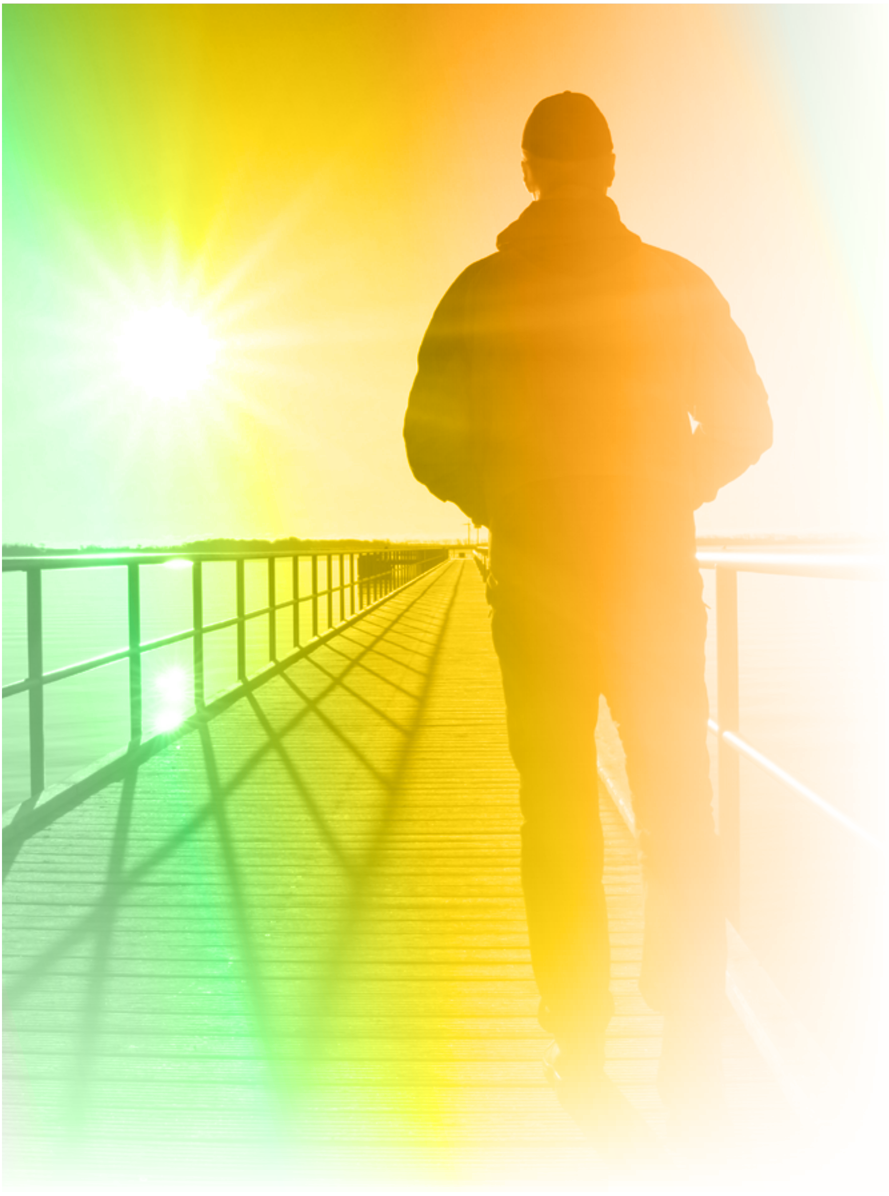

P3A

Psilocybine en fin de vie

Audace, Acceptabilité, Accès
