# Supplementary material for: Supporting Meaningful Choices: A Decision Aid for Individuals Facing Existential Distress and Considering Psilocybin-Assisted Therapy
Source: Healthcare (Basel). 2025 Sep 12;13(18):2290. doi: 10.3390/healthcare13182290 (PMC12469295; doi:10.3390/healthcare13182290)
Supplement: Supplementary file 1 [file healthcare-13-02290-s001.zip › Supplementary File S6. Decision Aid_French_Bélanger et al..pdf]

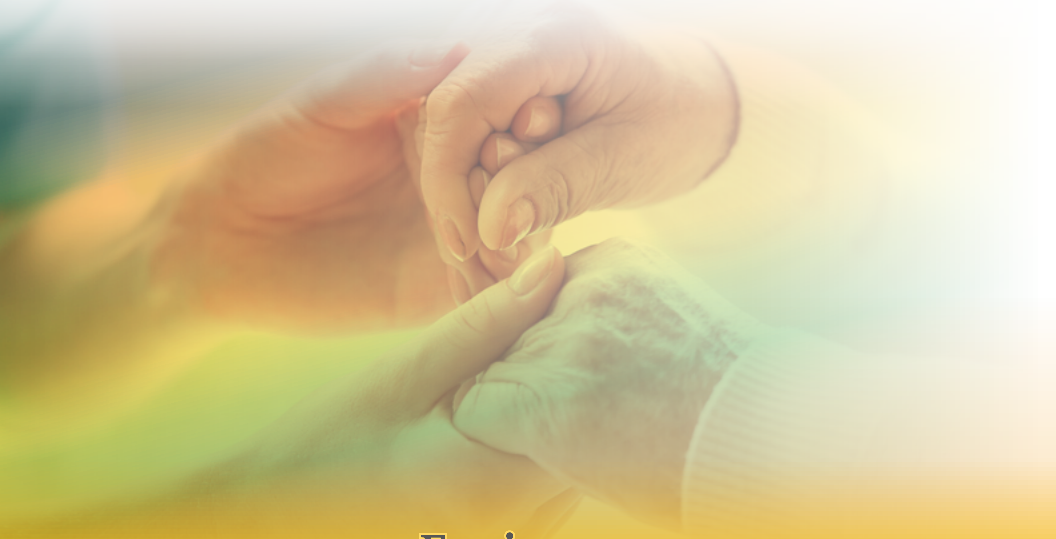

Envisager la THÉRAPIE assistée par *PSILOCYBINE* pour soulager la détresse existentielle

Outil d'aide à la décision

Cet outil s'adresse à vous si :

| vous êtes désespéré(e) en raison d'une maladie grave

| vous avez tenté plusieurs traitements pour soulager cette détresse

| vous voulez en savoir plus sur les traitements disponibles pour soulager cette détresse

| vous voulez en savoir plus sur la thérapie assistée par psilocybine.

Cette brochure a été réalisée par Ariane Bélanger, sous la supervision du professeur Michel Dorval, dans le cadre de sa maitrise en sciences pharmaceutiques à l'Université Laval.

ÉQUIPE DE RÉDACTION :

- Ariane Bélanger, B.Inf.
- Michel Dorval, Ph.D.
- Sue-Ling Chang, M.Sc.
- Florence Moureaux, patiente partenaire
- Jean-François Stephan, M.D.
- Diane Tapp, Ph.D.

PARTENAIRES FINANCIERS :

- Équipe de Recherche Michel-Sarrazin en Oncologie psychosociale et Soins palliatifs (ERMOS)
- Chaire de recherche en soins palliatifs de l'Université Laval
- Fonds d'enseignement et recherche (FER) de la Faculté de pharmacie de l'Université Laval

Nous remercions chaleureusement toutes les personnes ayant contribué au développement de cette brochure en commentant les versions antérieures. Ces contributions ont été recueillies dans le cadre d'un projet de recherche approuvé par le Comité d'éthique de la recherche du CHU de Québec-Université Laval (# 2024-7116).

Créé par Ariane Bélanger, Michel Dorval, Sue-Ling Chang
© Université Laval 2025. Tous droits réservés.

Aucune partie de ce document ne peut être modifiée et aucun élément ne peut être utilisé hors de son contexte sans permission écrite. Pour de l'information supplémentaire, adressez-vous à p3a@crchudequebec.ulaval.ca

Veuillez citer ce document comme suit : *Bélanger A, Dorval M, Chang SL. (2025) Envisager la thérapie assistée par psilocybine pour soulager la détresse existentielle : Outil d'aide à la décision. Université Laval, Québec.*

P3A
Psilocybine en fin de vie
Audace, Acceptabilité, Accès

UNIVERSITÉ LAVAL

Envisager la THÉRAPIE assistée par *PSILOCYBINE*

Pour soulager la détresse existentielle

Développez vos connaissances sur la détresse existentelle et les traitements possibles 5

1.1 Qu'est-ce que la détresse existentielle ? 5

1.2 Quels sont les traitements pour la détresse existentielle ? 6

Développez vos connaissances sur la thérapie assistée par psilocybine 8

2.1 Qu'est-ce que la psilocybine ? 8

2.2 Qu'est-ce que la thérapie assistée par psilocybine ? 9

2.3 Quels sont les bénéfices ? 12

2.4 Quels sont les risques ? 13

2.5 Quelles sont les contre-indications ? 15

2.6 Quelles sont les interactions médicamenteuses ? 16

Réfléchissez à ce qui est le plus important 17

Êtes-vous prêt(e) à faire votre choix ? 20

Ressources documentaires 24

Bibliographie 26

DÉVELOPPEZ vos connaissances sur la détresse existentielle et les traitements possibles

1.1 | Qu'est-ce que la détresse existentielle?

Apprendre qu'on a une maladie grave peut affecter nos émotions, nos relations sociales et notre vie spirituelle, créant un sentiment appelé détresse existentielle. Environ un tiers des personnes atteintes d'un cancer avancé ou d'une autre maladie grave au Canada ressentent des symptômes de détresse existentielle, d'anxiété et de dépression.

Les symptômes incluent :

- Un sentiment de découragement, de désespoir, de perte de sens et d'impuissance
- Une perte de sens à la vie
- La peur de mourir ou le désir de mourir
- Des symptômes de dépression et d'anxiété

> *Il est important de parler de vos symptômes avec un professionnel de la santé, **car il peut être difficile de distinguer un épisode dépressif temporaire de la détresse existentielle.** Les professionnels de la santé peuvent vous aider à comprendre vos symptômes et à trouver le meilleur traitement.*

1.2 | Quels sont les traitements disponibles pour la détresse existentielle

Il existe différents traitements pour soulager la détresse existentielle. Voici un aperçu des options disponibles.

Les traitements conventionnels :

- **Médicaments :** Certains médicaments, comme les anxiolytiques et les antidépresseurs, peuvent être prescrits pour soulager l'anxiété et la dépression qui accompagnent souvent la détresse existentielle. Ces médicaments doivent être prescrits et surveillés par un professionnel de la santé compétent.
- **Psychothérapie :** Certains types de psychothérapies, comme la thérapie cognitive-comportementale et la thérapie existentielle, peuvent aider les personnes à explorer leurs sentiments et à trouver un sens à leur vie malgré la maladie.
- **Accompagnement spirituel :** Parler avec un intervenant spirituel ou psychosocial peut aider les personnes à trouver du réconfort face à la maladie en tenant compte de leurs valeurs et de leurs croyances.

Les thérapies assistées par psychédéliques :

- **Thérapie assistée par psilocybine :** La psilocybine, un composé trouvé dans certains champignons, en combinaison avec une psychothérapie, peut aider les personnes avec une détresse existentielle. Cette approche sera expliquée plus en détails dans cet outil.
- **Thérapie assistée par kétamine :** La kétamine, un médicament utilisé pour l'anesthésie, est également utilisée pour son potentiel à aider les personnes en fin de vie. Elle a montré son efficacité pour traiter la dépression résistante, une condition souvent liée à la détresse existentielle.

Les traitements complémentaires :

- **Méditation et yoga :** Des approches comme la méditation et le yoga peuvent aider à réduire le stress et à améliorer le bien-être général.

Ces traitements peuvent être utilisés seuls ou combinés, selon les besoins et les préférences de chaque personne. Il est important d'en discuter avec un professionnel de la santé compétent pour déterminer les options les plus adaptées à votre situation.

Dans la prochaine section, nous explorerons plus en détail la thérapie assistée par psilocybine, afin de vous aider à déterminer si cette approche pourrait être une option pour soulager votre détresse existentielle.

DÉVELOPPEZ vos connaissances sur la thérapie assistée par psilocybine

Dans cette section, nous abordons ce que vous devez savoir sur la psilocybine, la thérapie assistée par psilocybine, ses bénéfices, ses risques, ses contre-indications et ses interactions médicamenteuses.

2.1 | Qu'est-ce que la psilocybine

La psilocybine est une substance naturelle présente dans certains champignons, souvent appelés « champignons magiques ». Une fois dans le corps, elle est transformée en une substance qui agit sur le cerveau. Elle stimule une molécule appelée sérotonine, qui joue un rôle important dans la gestion des émotions et de l'humeur.

Quand elle est utilisée dans un cadre thérapeutique, la psilocybine peut temporairement changer la façon dont le cerveau fonctionne, en rendant certaines parties plus connectées entre elles. Cela peut permettre de voir les choses différemment ou d'explorer de nouvelles façons de comprendre ses émotions.

Certaines personnes rapportent qu'elles se sentent plus sereines ou qu'elles trouvent du sens après leur expérience de psilocybine. Toutefois, ces effets varient beaucoup d'une personne à l'autre. Il n'y a aucune garantie que la psilocybine vous apportera ces bienfaits.

La thérapie assistée par psilocybine est un traitement encore à l'étude, utilisé uniquement dans des cas spécifiques. Au Canada, elle est accessible par le **Programme d'accès spécial** de Santé Canada, destiné aux patients atteints de maladies graves. Son accès est possible lorsque les autres traitements n'ont pas fonctionné ou ne conviennent pas.

Dans la section suivante, nous expliquerons comment se déroule une thérapie assistée par psilocybine.

2.2 | Qu'est-ce que la thérapie assistée par psilocybine

Avant de commencer la thérapie, une évaluation médicale est requise afin de vérifier si la thérapie est sécuritaire pour vous.

La thérapie assistée par psilocybine consiste à prendre une dose modérée de psilocybine tout en suivant une psychothérapie. Elle se déroule en trois étapes importantes 1) la préparation, 2) la prise de psilocybine, et 3) l'intégration. Ces étapes sont essentielles pour le succès de la thérapie et affectent ses résultats.

1 | La première étape, **c'est la préparation**

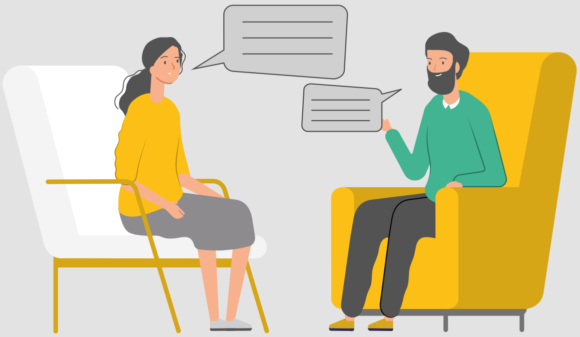

Cette étape vise à établir une relation de confiance avec un professionnel de la santé qui sera votre thérapeute et qui guidera la séance de la prise de psilocybine. Également, votre détresse existentielle sera explorée de différentes façons pour définir le but du traitement. Les rencontres préparatoires visent à comprendre vos attentes et à vous préparer à vivre le traitement en toute sécurité en vous fournissant les informations nécessaires pour la prochaine étape.

2 | La deuxième étape, **c'est la séance de la prise de psilocybine**

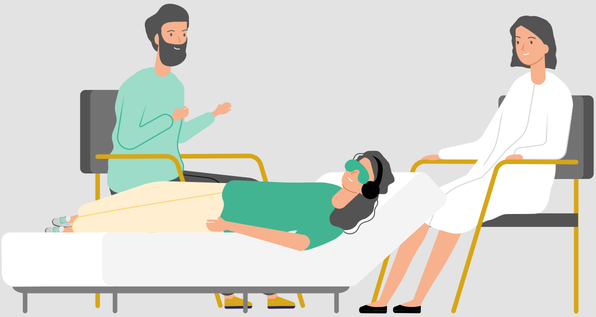

Cette séance se déroule dans un endroit confortable et apaisant, adapté à la thérapie. Dans certains cas, cela peut se faire à votre domicile. Vous serez allongé(e) avec un masque sur les yeux et des écouteurs pour écouter de la musique spécialement choisie pour vous aider à ressentir vos émotions et à favoriser l'introspection. Deux thérapeutes seront présents avec vous pendant la séance : le médecin que vous avez vu à la première étape et un autre professionnel de la santé. La séance dure environ 6 à 8 heures au cours desquelles la période avec le masque sur les yeux et la musique peut être entrecoupée de moment d'échanges avec les thérapeutes. Les thérapeutes sont présents pour accueillir et discuter de ce que vous ressentez.

3 | La troisième étape, **c'est l'intégration**

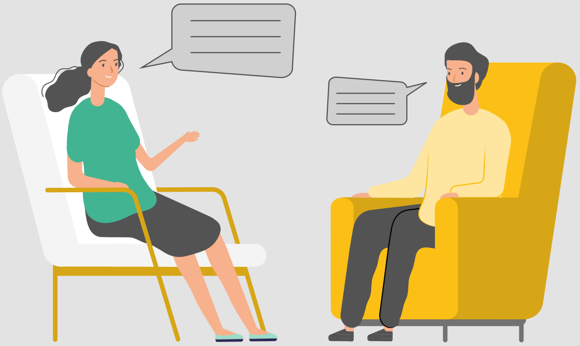

Cette étape consiste à discuter avec le thérapeute de ce que vous avez vécu lors de la séance de psilocybine. Cette période ressemble à une psychothérapie classique et peut se dérouler en une ou plusieurs séances. L'objectif principal est de vous aider à donner un sens à ce que vous avez vécu pendant la séance de prise de psilocybine. L'intégration vise également à vous aider à appliquer ces changements dans votre vie quotidienne. Pour certaines personnes, cela peut prendre plusieurs mois.

› TABLE DES MATIÈRES

2.3 | Quels sont les bénéfices possibles de la thérapie assistée par psilocybine?

Des informations provenant de récentes études cliniques montrent qu'une seule prise de psilocybine combinée à une psychothérapie peut avoir les effets suivants :

- Une diminution rapide et significative des symptômes de dépression et d'anxiété.
- Une amélioration des sentiments liées au découragement, au manque d'espoir ou à la perte de sens.
- Une diminution de la peur de mourir et une meilleure acceptation de la mort.
- Une amélioration de la qualité de vie et du bien-être psychologique et spirituel.

> ***Selon certaines études, ces effets pourraient durer au moins 6 mois, après une seule prise de psilocybine.***

› TABLE DES MATIÈRES

2.4 | Quels sont les risques possibles de la thérapie assistée par psilocybine?

Effets physiques	Effets psychologiques
**Effets les plus fréquents (>5%)**	
Augmentation de la tension artérielle (pression du sang)<br>Augmentation de la fréquence cardiaque<br>Céphalée (maux de tête)<br>Nausée (envie de vomir)	Anxiété (se sentir très inquiet)<br>Confusion (avoir du mal à comprendre ce qui se passe)
**Effets moins fréquents (<5%)**	
Fatigue<br>Migraine (maux de tête forts)<br>Vomissement<br>Inconfort physique (se sentir mal dans son corps)	Expériences difficiles (aussi appelées *bad trip*)<br>Inconfort psychologique (se sentir mal dans sa tête)<br>Crise spirituelle (remise en question de valeurs importantes)<br>Complications psychiatriques (aggravation de certains troubles mentaux)

Tableau inspiré de MacCallum et al.,2022, *Frontiers in Psychiatry.*

Ces effets secondaires potentiels sont connus grâce à des études faites dans un cadre contrôlé (avec beaucoup de surveillance). Dans des contextes moins supervisés, ces effets pourraient être plus fréquents.

Parfois, la psilocybine peut provoquer des moments désagréables, comme de l'anxiété ou un grand inconfort. Ces expériences, souvent appelées «bad trips», ne durent pas longtemps. Certaines personnes peuvent ressentir une déception lorsque la thérapie n'apporte pas les résultats escomptés, ce qui peut parfois aggraver leur état initial.

Les personnes ayant des antécédents personnels ou familiaux de troubles de santé mentale doivent consulter un professionnel de la santé avant de commencer ce type de thérapie. Bien que rare, un

risque de complication de santé mentale, comme des symptômes psychotiques, existe.

Pour minimiser ces risques, les séances de préparation sont très importantes. Elles permettent de comprendre le processus et de se préparer à d'éventuelles expériences difficiles. Pendant la thérapie, les professionnels assurent une supervision constante, rendant ce traitement aussi sécuritaire que possible.

Même si une expérience peut être difficile sur le moment, elle peut être bénéfique avec le travail d'intégration fait lors de la psychothérapie. Ce suivi aide les personnes à tirer le maximum de leur expérience.

2.5 | Quelles sont les contre-indications ?

Il n'y a pas de contre-indications strictes à la thérapie assistée par psilocybine. Cela signifie que, pour la plupart des personnes, il est possible d'en discuter avec un médecin pour décider si c'est une bonne option pour elles-mêmes. Les raisons principales pour ne pas utiliser cette thérapie sont liées à certains problèmes de santé. Par exemple :

- **Problèmes cardiaques graves ou non maîtrisés :** Comme de l'insuffisance cardiaque, de l'hypertension non contrôlée ou de l'arythmie sévère.
- **Insuffisance hépatique :** Des problèmes graves au foie.
- **Certaines atteintes cérébrales :** Des problèmes spécifiques au cerveau.
- **Antécédents de psychoses :** Par exemple, des troubles comme la schizophrénie et la maladie affective bipolaire.
- Il est aussi déconseillé d'utiliser cette thérapie pendant la **grossesse** et l'**allaitement**, car il n'y a pas assez de preuves scientifiques sur les risques possibles.

> ***Pour savoir si cette thérapie peut vous convenir, il est important de discuter des risques et des bénéfices avec votre médecin. Cette décision se prend en collaboration avec votre médecin, qui évaluera votre état de santé et vous aidera à prendre la meilleure décision pour vous.***

› TABLE DES MATIÈRES

2.6 | Quelles sont les interactions médicamenteuses possibles ?

Avant d'entreprendre une thérapie assistée par psilocybine, il est important de discuter avec un professionnel de la santé compétent des médicaments que vous prenez déjà. Certains médicaments peuvent interagir avec la psilocybine, et votre médecin pourra vous conseiller sur les ajustements nécessaires.

Pour les traitements contre le cancer, comme la chimiothérapie et la radiothérapie, les études actuelles ne montrent pas de preuves d'interactions avec la psilocybine. Dans les études cliniques, ces traitements ont été continués sans problèmes apparents liés à la psilocybine, mais il est toujours prudent d'en parler avec votre médecin.

> ***Consultez votre médecin pour comprendre les risques potentiels liés aux interactions médicamenteuses avec la thérapie assistée par psilocybine.***

› TABLE DES MATIÈRES

RÉFLÉCHISSEZ à ce qui est le plus important pour vous

Utilisez l'échelle des pages suivantes pour vous aider à évaluer vos valeurs personnelles en ce qui concerne la thérapie assistée par psilocybine. Sur cette échelle, plus votre évaluation se situe du côté gauche, plus vous êtes en faveur de la thérapie assistée par psilocybine. Par exemple, si vous accordez beaucoup d'importance à éviter les émotions difficiles, cela pourrait influencer votre position sur l'échelle. Discutez de vos réponses et de vos réflexions avec votre professionnel de la santé pour prendre une décision éclairée.

1 | Qualité de vie

Les symptômes affectent ma qualité de vie et mes activités de la vie quotidienne	❑ Me correspond totalement	❑ Me correspond un peu	❑ Ne sais pas	❑ Me correspond un peu	❑ Me correspond totalement	Je suis capable de fonctionner et ma qualité de vie est acceptable

2 | Peur de la mort

La mort me fait peur et me crée de la détresse	❑ Me correspond totalement	❑ Me correspond un peu	❑ Ne sais pas	❑ Me correspond un peu	❑ Me correspond totalement	Je n'ai pas peur de la mort et l'anxiété ne me cause pas de détresse

3 | Autres traitements

J'ai tenté d'autres traitements qui se sont avérés inefficaces pour soulager mes symptômes	❑ Me correspond totalement	❑ Me correspond un peu	❑ Ne sais pas	❑ Me correspond un peu	❑ Me correspond totalement	Je n'ai pas tenté d'autres traitements pour soulager mes symptômes

4 | Tolérance aux émotions

Je suis prêt(e) à vivre des émotions qui peuvent être difficiles dans une visée thérapeutique	❑ Me correspond totalement	❑ Me correspond un peu	❑ Ne sais pas	❑ Me correspond un peu	❑ Me correspond totalement	Je ne peux pas supporter plus d'émotions difficiles, j'ai besoin d'un soulagement immédiat

› TABLE DES MATIÈRES

5 | Effets secondaires

Mes symptômes sont pires que les possibles effets secondaires de la psilocybine	❑ Me correspond totalement	❑ Me correspond un peu	❑ Ne sais pas	❑ Me correspond un peu	❑ Me correspond totalement	Je pense que les effets secondaires seront plus difficiles que mes symptômes présents

6 | Engagement thérapeutique

Je suis prêt(e) à faire les démarches nécessaires pour obtenir l'accès à la thérapie	❑ Me correspond totalement	❑ Me correspond un peu	❑ Ne sais pas	❑ Me correspond un peu	❑ Me correspond totalement	Je ne suis pas prêt(e) à faire les démarches nécessaires, car cela demande trop de temps

7 | Ouverture à la spiritualité

Je suis ouvert(e) à développer ma spiritualité et à me laisser guider par une expérience introspective	❑ Me correspond totalement	❑ Me correspond un peu	❑ Ne sais pas	❑ Me correspond un peu	❑ Me correspond totalement	Je ne suis pas ouvert(e) au développement spirituel et je ne me sens pas en mesure de me laisser guider par une expérience introspective

8 | Intention d'entreprendre une thérapie

Je suis prêt(e) à m'impliquer dans une thérapie de plusieurs séances qui nécessite du temps et de l'introspection	❑ Me correspond totalement	❑ Me correspond un peu	❑ Ne sais pas	❑ Me correspond un peu	❑ Me correspond totalement	J'ai besoin d'un soulagement immédiat, je ne veux pas m'impliquer dans une thérapie.

Êtes-vous prêt(e) à faire votre CHOIX ?

Questions les plus posées (FAQ) :

Est-ce que la thérapie assistée par psilocybine s'adresse à tout le monde ?

Chez certaines personnes, la thérapie assistée par psilocybine peut ne pas donner les résultats désirés, provoquant parfois de la déception, même du désespoir. Cette thérapie requiert un engagement important de la part de la personne. Il est crucial de discuter ouvertement avec votre professionnel de la santé, de vos attentes et de vos intentions concernant ce traitement.

Qu'est-ce que je peux attendre de la thérapie assistée par psilocybine ?

Le traitement permet d'explorer des pensées et émotions que l'on n'arrive pas à exprimer normalement. Vous pourriez ressentir des changements dans la manière de voir les choses. Les effets peuvent varier d'une personne à l'autre. Les études montrent que beaucoup de gens ressentent moins d'anxiété et de dépression après le traitement, mais cela ne fonctionne pas pour tout le monde.

Y a-t-il un risque de dépendance ?

La psilocybine n'entraîne pas de dépendance physique et présente un faible niveau de toxicité. Son utilisation sous surveillance médicale est considérée comme sécuritaire.

Quel est le coût du traitement ?

Au Québec, le traitement est remboursé par la Régie de l'assurance maladie du Québec (RAMQ) lorsqu'administré par des médecins ou dans un centre hospitalier. Si le traitement est effectué en dehors de ces établissements, les frais du médecin sont couverts, mais il pourrait y avoir des coûts pour la personne.

Comment avoir accès au traitement ?

Il est possible d'accéder au traitement par le biais du Programme d'accès spécial de Santé Canada. La demande doit être faite par un médecin. Vous devriez discuter avec votre médecin pour savoir s'il

serait disposé à vous accompagner dans cette démarche ou, dans le cas contraire, vous référer pour obtenir des informations. Si nécessaire, vous pouvez également contacter TheraPsil, une organisation à but non lucratif qui aide les Canadiens éligibles à accéder à la thérapie assistée par psilocybine. Cependant, même si votre médecin soumet une demande au Programme d'accès spécial, l'obtention de l'autorisation n'est pas garantie. **Il est important de noter qu'il s'agit d'un traitement d'exception.**

Qu'est-ce que le microdosage ? Est-ce une alternative ?

Le microdosage de la psilocybine consiste à prendre environ 1/10 à 1/20 du dosage thérapeutique habituel presque tous les jours pour soulager les symptômes d'anxiété et de dépression. Bien que de plus en plus de Canadiens adoptent cette pratique, la psilocybine demeure illégale, et les études sur le microdosage ne fournissent pas de conclusions définitives. Aucune preuve actuelle ne soutient cette méthode, et elle n'est donc pas recommandée.

À la suite de ma réflexion, j'arrive à la conclusion suivante :

- ❏ Je suis certain(e) de vouloir considérer la thérapie assistée par psilocybine.
- ❏ Je pense que d'autres traitements pourraient être plus appropriés pour moi :
  - Médicaments
  - Psychothérapie
  - Accompagnement spirituel
  - Thérapies complémentaires (méditation, yoga)
- ❏ Je ne sais pas quel est le traitement qui me conviendrait le mieux, mais je penche vers l'option suivante :

> *Assurez-vous de discuter avec votre professionnel de la santé pour explorer la possibilité de combiner plusieurs traitements pour un meilleur soutien global.*

Notes et réflexions :

› TABLE DES MATIÈRES

Si vous n'êtes pas certain(e) que la thérapie assistée par psilocybine soit une option qui peut vous convenir, le tableau suivant peut vous aider à identifier les aspects qui rendent votre décision difficile. Vous pouvez identifier ce qui s'applique à vous pour trouver des moyens de répondre à vos besoins.

Ce qui rend ma décision difficile	Des actions possibles
Je n'ai pas assez d'informations sur les autres traitements disponibles pour la détresse existentielle et leurs risques et avantages.	| Faire une liste de mes questions<br>| Chercher des ressources fiables que je peux utiliser pour trouver l'information désirée (ex. : professionnel de la santé, études scientifiques, essais cliniques, internet, organisme à but non lucratif, etc.)
Je ne suis pas certain(e) de souffrir de détresse existentielle, d'anxiété ou de dépression.	| Parler de mes symptômes avec un professionnel de la santé
Je ne suis pas certain(e) de savoir si ce sont les avantages ou les désavantages de la thérapie assistée par psilocybine qui comptent le plus pour moi	| Parler avec un professionnel de la santé pour comprendre comment d'autres personnes avec des symptômes similaires ont pris leur décision.<br>| Lire des témoignages d'autres personnes ayant choisi la thérapie assistée par psilocybine.
Je manque de soutien et de ressources pour prendre une décision éclairée.	| Demander l'avis d'un professionnel de la santé et discuter avec une personne en qui j'ai confiance.

Partagez vos réponses avec votre médecin.

RESSOURCES DOCUMENTAIRES

Ressources pour les professionnels de la santé (Ressources Web)

Ressource	Description
TheraPsil	Organisation à but non lucratif canadienne qui aide les professionnels de la santé à naviguer dans le processus d'accès à la thérapie assistée par la psilocybine. therapsil.ca
Multidisciplinary Association for Psychedelic Studies (MAPS)	Organisation de recherche fournissant des informations et des ressources sur les psychédéliques en thérapie. maps.org
Psychedelic Medicine Podcast – Dre Lynn Morski	Plusieurs balados dédiés à discuter des usages thérapeutiques des psychédéliques, y compris la psilocybine, avec des experts du domaine médicale. plantmedicine.org
The American Trip – Ido Hartogsohn	Un livre qui explore le concept de « set and setting » et son impact sur les expériences psychédéliques. Disponible en librairie.
Le reportage de Découvertes du 12 novembre 2023 sur les psychédéliques	Présente une séance de thérapie assistée par la psilocybine au Québec, avec témoignages et encadrement clinique, très utile pour les patients. Épisode du dimanche 12 novembre 2023

Publications et Études

1 | **Griffiths, R. R., et al. (2016)** : *Psilocybin produces substantial and sustained decreases in depression and anxiety in patients with life-threatening cancer: A randomized double-blind trial. Journal of Psychopharmacology, 30(12), 1181-1197. PubMed.*

2 | **Ross, S., et al. (2016)** : *Rapid and sustained symptom reduction following psilocybin treatment for anxiety and depression in patients with life-threatening cancer: A randomized controlled trial. Journal of Psychopharmacology, 30(12), 1165-1180. PubMed.*

3 | **Grob, C. S., et al. (2011)** : *Pilot study of psilocybin treatment for anxiety in patients with advanced-stage cancer. Archives of General Psychiatry, 68(1), 71-78. PubMed.*

4 | **MacCallum, C. A., et al. (2022)** : *Therapeutic use of psilocybin: Practical considerations for dosing and administration. Frontiers in Psychiatry, 13, 1040217. PubMed.*

5 | **Nichols, D.E. (2016)** : *Psychedelics. Pharmacological Reviews, 68(2), 264-355. PubMed.*

6 | **National Cancer Institute. (2023)** : *Ongoing Clinical Trials on Psilocybin in Palliative Care. National Cancer Institute.*

7 | **Griffiths, R. R., et al. (2023)** : *Long-term Follow-up of Psilocybin- Assisted Psychotherapy for Psychiatric and Existential Distress in Patients with Life-threatening Cancer. Journal of Psychopharmacology. PubMed.*

Ressources pour le public
(Ressources web)

Ressource	Description
TheraPsil	Information et soutien pour les patients canadiens cherchant à accéder à la thérapie assistée par la psilocybine. therapsil.ca
Psychedelic Support	Plateforme fournissant des informations et du soutien aux personnes qui explorent les traitements psychédéliques, y compris la psilocybine. psychedelic.support/resources/psilocybin-assisted-therapy-guide

Documentaires

1 | **Radzio-Canada - Au cœur d'une thérapie psychédélique** : Un reportage de Radio-Canada qui explore une thérapie assistée par la psilocybine. ici.radio-canada.ca/info/long-format/2025449/coeur-therapie-psychedelique-psilocybine

2 | **Dosed : The trip of a lifetime – Golden Teacher film** : Un documentaire qui explore les effets thérapeutiques de la psilocybine. dosedmovie.com (en anglais)

Balados

1 | **Radio-Canada – Voyage inédit au cœur des thérapies assistées par les psychédéliques** : Un balado de Radio-Canada qui explore l'utilisation médicale des psychédéliques. ici.radio-canada.ca/ohdio/balados/6108/ca-sexplique-balado-info-alexis-de-lancer/837309/drogue-therapie-champignons-lsd-psychedeliques-experience

2 | **France Culture - Explorations psychédéliques** : Une série de balados qui explore les utilisations médicinales des psychédéliques, y compris la psilocybine. radiofrance.fr/franceculture/podcasts/serie-explorations-psychedeliques

3 | **Plant Medicine Podcast** : Des balados qui discutent des substances psychoactives avec des experts, y compris des psychédéliques comme la psilocybine, et leurs applications thérapeutiques. Le contenu est plus poussé et pointu, mais reste accessible à ceux qui ont un grand intérêt pour les psychédéliques. plantmedicine.org (en anglais)

4 | **Huberman Lab - How Psilocybin Can Rewire Our Brain, Its therapeutic Benefits & its Risks** : Un épisode de podcast qui explore comment la psilocybine peut reconfigurer notre cerveau, ses bénéfices thérapeutiques et ses risques. hubermanlab.com/episode/how-psilocybin-can-rewire-our-brain-its-therapeutic-benefits-and-its-risks (en anglais)

5 | **Le balado «Psychedelic Therapy Frontiers» – produit par Numinus** : Conseils pour bien se préparer, vivre et intégrer une expérience avec la psilocybine. youtube.com/@numinusnetwork

Livres

1 | **Voyage aux confins de l'esprit – Michael Pollan (2018)** : Ce livre explore l'usage thérapeutique des psychédéliques, y compris la psilocybine, et leurs effets sur la santé mentale.

2| **The American Trip - Ido Hartosohn** : Un livre sur l'importance du «set and setting» dans les expériences psychédéliques (en anglais).

3| **Phantastica: ces substances interdites qui guérissent - Stéphanie Chayet (2020)** : Fondée sur l'expérience de l'auteure, ce livre questionne la distinction entre drogues et médicaments et montre les effets d'un recours aux psychédéliques.

BIBLIOGRAPHIE

Breitbart, William, and Harvey Chochinov. *Handbook of Psychiatry in Palliative Medicine 3rd Edition.* 2022. doi:10.1093/med/9780197583838.001.0001.

Chochinov, H. M., L. J. Kristjanson, W. Breitbart, S. McClement, T. F. Hack, T. Hassard, and M. Harlos. "Effect of Dignity Therapy on Distress and End-of-Life Experience in Terminally Ill Patients: A Randomised Controlled Trial." *Lancet Oncol* 12, no. 8 (Aug 2011): 753-62. https://doi.org/10.1016/S1470-2045(11)70153-X. https://www.ncbi.nlm.nih.gov/pubmed/21741309

Clarke, D. M., and D. W. Kissane. "Demoralization: Its Phenomenology and Importance." [In eng]. *Aust N Z J Psychiatry* 36, no. 6 (Dec 2002): 733-42. https://doi.org/10.1046/j.1440-1614.2002.01086.x

Davis, A. K., F. S. Barrett, D. G. May, M. P. Cosimano, N. D. Sepeda, M. W. Johnson, P. H. Finan, and R. R. Griffiths. "Effects of Psilocybin-Assisted Therapy on Major Depressive Disorder: A Randomized Clinical Trial." *JAMA Psychiatry* 78, no. 5 (May 1 2021): 481-89. https://doi.org/10.1001/jamapsychiatry.2020.3285. https://www.ncbi.nlm.nih.gov/pubmed/33146667

Griffiths, R. R., M. W. Johnson, M. A. Carducci, A. Umbricht, W. A. Richards, B. D. Richards, M. P. Cosimano, and M. A. Klinedinst. "Psilocybin Produces Substantial and Sustained Decreases in Depression and Anxiety in Patients with Life-Threatening Cancer: A Randomized Double-Blind Trial." *J Psychopharmacol* 30, no. 12 (Dec 2016): 1181-97. https://doi.org/10.1177/0269881116675513 - https://www.ncbi.nlm.nih.gov/pubmed/27909165

Grob, C. S., A. L. Danforth, G. S. Chopra, M. Hagerty, C. R. McKay, A. L. Halberstadt, and G. R. Greer. "Pilot Study of Psilocybin Treatment for Anxiety in Patients with Advanced-Stage Cancer." *Arch Gen Psychiatry* 68, no. 1 (Jan 2011): 71-8. https://doi.org/10.1001/archgenpsychiatry.2010.116. https://www.ncbi.nlm.nih.gov/pubmed/20819978

Johnson, M. W., W. A. Richards, and R. R. Griffiths. "Human Hallucinogen Research: Guidelines for Safety." *Journal of Psychopharmacology* 22 (6) (2008): 603-20. https://doi.org/10.1177/026988110809358

Kissane, D. W. , D. M. Clarke, and A.F Street. "Demoralization Syndrome-a Relevant Psychiatric Diagnosis for Palliative Care." [In eng]. *Journal of Palliative Care* 17, no. 1 (2001): 12-21.

Lee, W., C. Sheehan, R. Chye, S. Chang, A. Bayes, C. Loo, B. Draper, M. R. Agar, and D. C. Currow. "Subcutaneous Ketamine Infusion in Palliative Patients for Major Depressive Disorder (Skipmdd)-Phase Ii Single-Arm Open-Label Feasibility Study." *PLoS One* 18, no. 11 (2023): e0290876. https://doi.org/10.1371/journal.pone.0290876 - https://www.ncbi.nlm.nih.gov/pubmed/37963146

LeMay, K., and K. G. Wilson. "Treatment of Existential Distress in Life Threatening Illness: A Review of Manualized Interventions." *Clin Psychol Rev* 28, no. 3 (Mar 2008): 472-93. https://doi.org/10.1016/j.cpr.2007.07.013. https://www.ncbi.nlm.nih.gov/pubmed/17804130

MacCallum, C. A., L. A. Lo, C. A. Pistawka, and J. K. Deol. "Therapeutic Use of Psilocybin: Practical Considerations for Dosing and Administration." *Front Psychiatry* 13 (2022): 1040217. https://doi.org/10.3389/fpsyt.2022.1040217. https://www.ncbi.nlm.nih.gov/pubmed/36532184

Mitchell, A. J., M. Chan, H. Bhatti, M. Halton, L. Grassi, C. Johansen, and N. Meader. "Prevalence of Depression, Anxiety, and Adjustment Disorder in Oncological, Haematological, and Palliative-Care Settings: A Meta-Analysis of 94 Interview-Based Studies." *Lancet Oncol* 12, no. 2 (Feb 2011): 160-74. https://doi.org/10.1016/S1470-2045(11)70002-X - https://www.ncbi.nlm.nih.gov/pubmed/21251875

Oyetunji, A., C. Huelga, K. Bunte, R. Tao, and V. Bellman. "Use of Ketamine for Depression and Suicidality in Cancer and Terminal Patients: Review of Current Data." *AIMS Public Health* 10, no. 3 (2023): 610-26. https://doi.org/10.3934/publichealth.2023043 - https://www.ncbi.nlm.nih.gov/pubmed/37842268

Ross, S., A. Bossis, J. Guss, G. Agin-Liebes, T. Malone, B. Cohen, S. E. Mennenga, *et al.* "Rapid and Sustained Symptom Reduction Following Psilocybin Treatment for Anxiety and Depression in Patients with Life-Threatening Cancer: A Randomized Controlled Trial." *J Psychopharmacol* 30, no. 12 (Dec 2016): 1165-80. https://doi.org/10.1177/0269881116675512 - https://www.ncbi.nlm.nih.gov/pubmed/27909164

Vehling, S., D. W. Kissane, C. Lo, H. Glaesmer, T. J. Hartung, G. Rodin, and A. Mehnert. "The Association of Demoralization with Mental Disorders and Suicidal Ideation in Patients with Cancer." *Cancer* 123, no. 17 (Sep 1 2017): 3394-401. https://doi.org/10.1002/cncr.30749 - https://www.ncbi.nlm.nih.gov/pubmed/28472548

Vehling, S., Y. Tian, C. Malfitano, J. Shnall, S. Watt, A. Mehnert, A. Rydall, *et al.* "Attachment Security and Existential Distress among Patients with Advanced Cancer." *J Psychosom Res* 116 (Jan 2019): 93-99. https://doi.org/10.1016/j.jpsychores.2018.11.018 - https://www.ncbi.nlm.nih.gov/pubmed/30655000

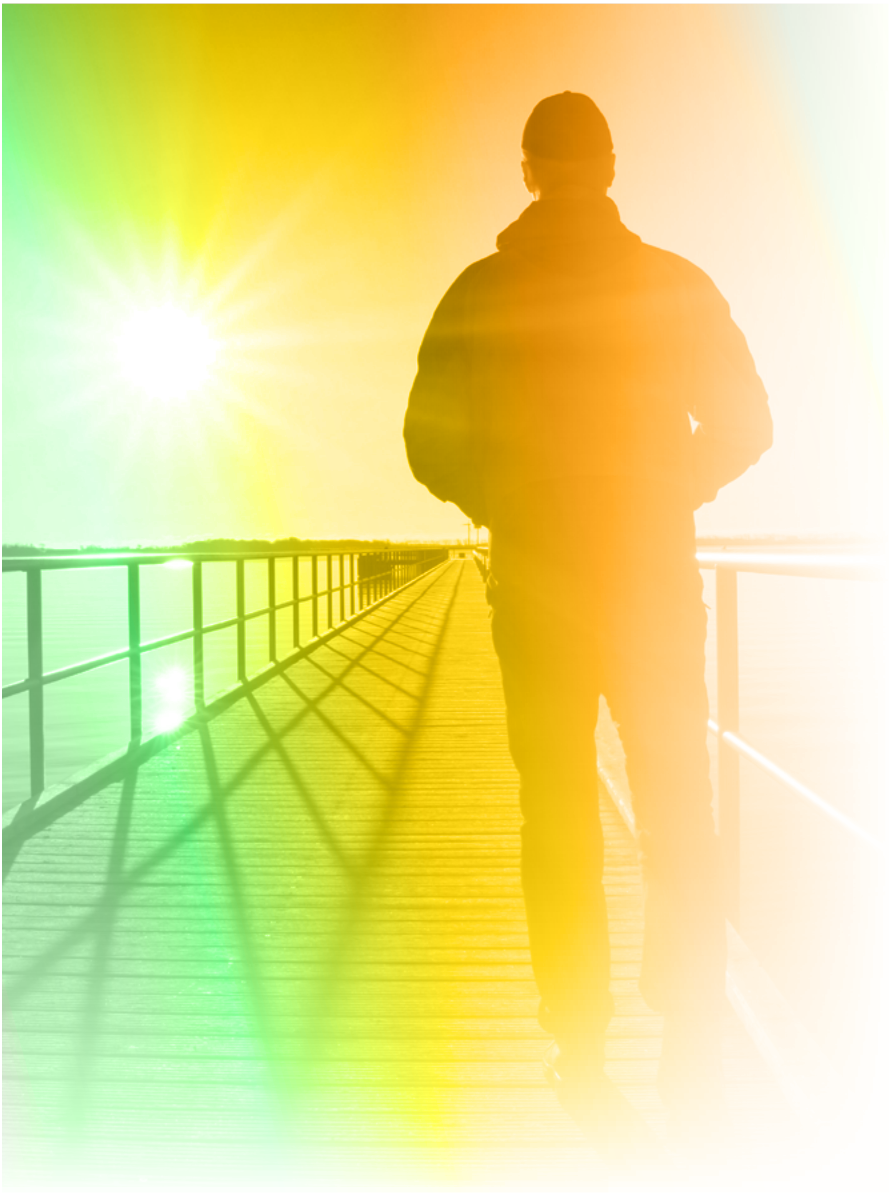

P3A

Psilocybine en fin de vie
Audace, Acceptabilité, Accès
